# Supplementary material for: Bayesian evaluation of diverging theories of episodic and affective memory distortions in dysphoria
Source: Nat Commun. 2024 Feb 13;15:1320. doi: 10.1038/s41467-024-45203-4 (PMC10864297; doi:10.1038/s41467-024-45203-4)
Supplement: Supplementary file 1 — Supplementary Information [file 41467_2024_45203_MOESM1_ESM.pdf]

SUPPLEMENTARY NOTE 1:
SUMMARY OF THREE PILOT STUDIES OF THE ROAM

Affective expression of autobiographical memories. In order to validate and refine the ROAM in its capability to assess episodic memory detail and to track the psychophysiological expression of emotion during autobiographical memory retrieval, we conducted three pilot studies with $N = 8$, $N = 5$, and $N = 8$. For the first pilot, we instructed participants to select five negative and five positive memories that could be important or trivial to them. While these instructions lead to some re-expression of emotionality assessed with fEMG, the responses to negative memories were not as strong as expected (according to visual inspection, **Supplementary Fig. 1a and b**). For the second pilot, we therefore asked participants to only select memories of events that were emotionally meaningful to them. Participants expressed strong physiological responses to negative memories consistent with our expectations (according to visual inspection, **Supplementary Fig. 1c and d**). For the final pilot, we added a neutral control condition. Further, we measured fEMG responses in two additional muscle regions (frontalis and depressor anguli oris[112]. A visual inspection of plots of the average time-course of each muscle per condition clearly showed that the frontalis and the depressor anguli oris did not distinguish better between negative, neutral, and positive memories than the corrugator and the zygomaticus (**Supplementary Fig. 2**). Therefore, frontalis and depressor anguli oris activity will not be further assessed in this study.

Episodic detail of autobiographical memories. In order to assess the episodic detail of each memory, we coded memories of the final pilot. We followed the coding instructions for autobiographical memories developed by Levine and colleagues[33,62]. However, we modified the codebook slightly by adding some examples and specific decision trees to increase inter-rater reliability and to adjust it to the ROAM. Specifically, two researchers coded the first three memories of each participant independently and we calculated the Intraclass Correlation Coefficient (ICC = 0.831) and Krippendorf's alpha ($\alpha_K = 0.825$) for the number of episodic details coded per memory by each rater. Even though interrater reliability was already high, there were minor inconsistencies. These were discussed and addressed in a revised coding manual. Using the refined coding scheme, two raters coded the following five memories of each participant independently (ICC = 0.946, $\alpha_K = 0.946$). Finally, two raters coded the last five memories of each participant (ICC = 0.937, $\alpha_K = 0.936$). For all pilot statistics regarding episodic detail, we averaged the coding of the two independent raters.

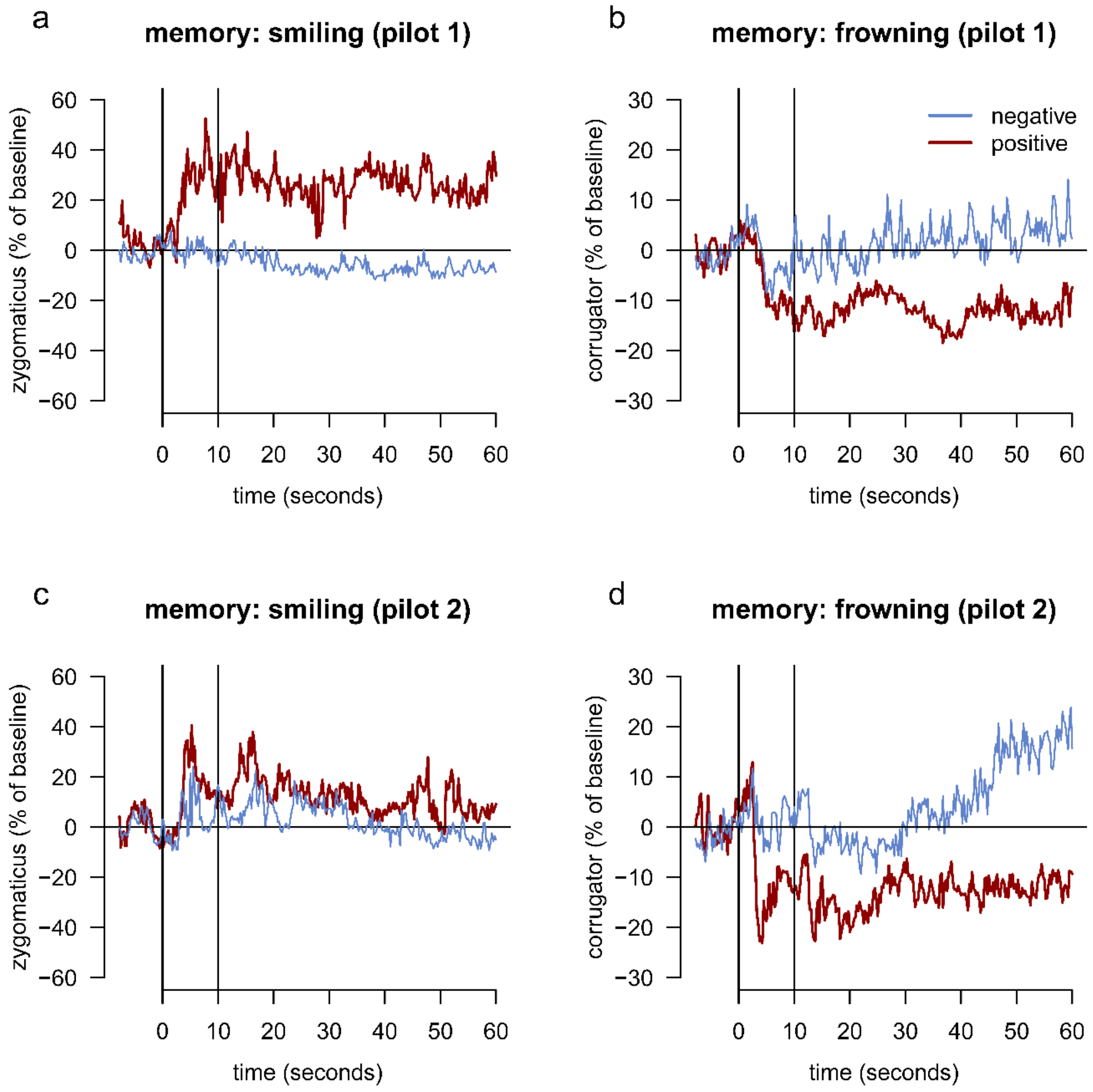

Supplementary Fig. 1 The psychophysiological expression of affect in pilot 1 and pilot 2. a and **b** present zygomaticus and corrugator activity during the retrieval of positive and negative memories in pilot 1 with $N = 8$ participants. **c** and **d** present zygomaticus and corrugator activity in pilot 2 with $N = 5$ participants. Muscle activity is presented as percentage change from baseline (4 s). The first vertical line indicates the onset of the memory cues (0 s), the second vertical line indicates the offset of the memory cues (10 s). For the remaining 50 seconds participants kept silently reliving the memory. In contrast to pilot 3 and the registered report (with 1000ms segments), data of pilot 1 and 2 were down-sampled to 250ms segments.

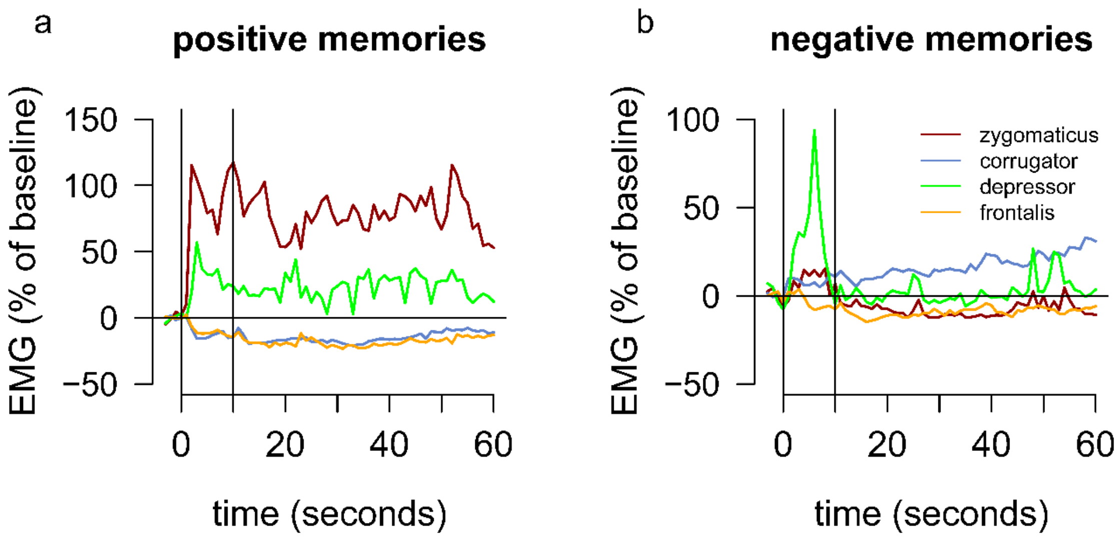

Supplementary Fig. 2 Comparison of potential muscles to measure affective responses in the ROAM (pilot 3 with *N* = 8 participants). a and **b** present muscle activity during the retrieval of positive and negative autobiographical memories, respectively. Muscle activity is presented as percentage change from baseline (4 s). The first vertical line indicates the onset of the memory cues (0 s), the second vertical line indicates the offset of the memory cues (10 s). For the remaining 50 seconds participants kept silently reliving the memory.

SUPPLEMENTARY NOTE 2: RESULTS OF THE FINAL PILOT STUDY OF THE ROAM

Note:
This supplementary note was part of the Methods section of the in principle accepted Stage 1 Registered Report.

Episodic detail of autobiographical memories. Two raters independently coded the amount of internal episodic detail of each memory (for more detail, see **Methods: episodic detail – coding**). The interrater reliability of the coding of episodic detail was high (Intraclass Correlation Coefficient ICC = 0.937 and Krippendorf's alpha α_K = 0.936, see **Supplementary Note 1** for more detail). We investigated descriptive statistics to evaluate whether the coded episodic detail would be suitable to investigate episodic detail in the ROAM. A multilevel model with a fixed effect for condition and a random intercept for participant showed that participants retrieved around twenty episodic details that were internal to the retrieved memory (estimated means negative: M = 20.863, SE = 1.741, positive: M = 22.238, SE = 1.741, neutral: M = 16.431, SE = 1.781). Further, we calculated the intraclass correlation (ICC) for episodic detail in a multilevel model in which episodic detail across all conditions is predicted by only a fixed intercept and a random intercept of participant. The intraclass correlation (ICC = .393) indicated that 39% of the variance of episodic detail results from between-participants variance and 61% results from within-participants variance. To conclude, our pilot data showed that episodic detail can be reliably coded by independent raters, and it captures inter- as well as intraindividual differences in how memories are retrieved.

Affective expression of autobiographical memories. We tested whether corrugator and zygomaticus response magnitudes allow the assessment of affective responses to positive and negative autobiographical memories. fEMG data of the pilot are displayed in **Supplementary Fig. 3a and 3b**. It can be observed that responses were relatively consistent and did not habituate with time. We averaged the data for the entire 1-minute retrieval segment and performed our analyses on these segments. We evaluated evidence for the hypotheses that zygomaticus activity is higher during the recall of positive memories than during their preceding baseline (manipulation check 1.1 – H1, BF_{1c} = 422.414, $PostP_1$ = . 968), that zygomaticus activity is lower during recall of positive memories than during baseline (manipulation check 1.1 – H2, BF_{H2c} = 0.002, $PostP_2$ = .002) and that zygomaticus activity is similar during recall of positive memories and baseline (manipulation check 1.1 – H0, BF_{0c} = 0.060, $PostP_0$ = .020). The results provided strong evidence that the zygomaticus is more active

during the recall of positive memories than during baseline. To test whether this response is specific for positive memories, we evaluated evidence for the hypotheses that the baseline-corrected zygomaticus response is higher when remembering positive memories than when remembering neutral memories (manipulation check 1.2 – H1, BF_{1c} = 57.948, $PostP_1$ = .825), that the zygomaticus response is lower for positive memories than for neutral memories (manipulation check 1.2 – H2, BF_{2c} = 0.017, $PostP_2$ = .014), and that the zygomaticus response is similar for positive and neutral memories (manipulation check 1.2 – H0, BF_{0c} = 0.382, $PostP_0$ = .161). These results provided strong evidence that participants smile more when reliving positive than when reliving neutral memories.

We evaluated evidence for the hypotheses that corrugator activity is higher during the recall of negative memories than during baseline (manipulation check 1.1 – H1, BF_{1c} = 44.911, $PostP_1$ = .842), that corrugator activity is lower during recall of negative memories than during baseline (manipulation check 1.1 – H2, BF_{2c} = 0.022, $PostP_2$ = .019), and that corrugator activity is similar during the recall of negative memories and during baseline (manipulation check 1.1 – H0, BF_{0c} = 0.324, $PostP_0$ = .139). The results provided strong evidence that the corrugator is more active during the recall of negative memories than during baseline. To test whether this response is specific for negative memories, we evaluated evidence for the hypotheses that the baseline-corrected corrugator response is higher when remembering negative memories than when remembering neutral memories (manipulation check 1.2 – H1, BF_{1c} = 18.825, $PostP_{11}$ = .680), that the corrugator response is lower when remembering negative memories than when remembering neutral memories (manipulation check 1.2 – H2, BF_{2c} = 0.053, $PostP_2$ = .036), and that the corrugator response is similar for negative and neutral memories (manipulation check 1.2 – H0, BF_{0c} = 0.792, $PostP_0$ = .284). These results provided evidence that participants frowned more when reliving negative than when reliving neutral memories.

In sum, the findings regarding zygomaticus and corrugator activity during memory retrieval provided compelling evidence that the ROAM allows to measure the psychophysiological expression of affect during autobiographical memory retrieval.

Relationship between episodic detail and affective response. We tested whether within-participant variations in episodic detail (i.e. participant-centred episodic detail) predicts affective responses to memories. For positive memories, we compared evidence for a positive relationship between episodic detail and zygomaticus responses (H1: $\gamma > 0$, BF_{1c} = 18.209, $PostP_1$ = .700), for a negative relationship (H2: $\gamma < 0$, BF_{2c} = .055, $PostP_2$ = .028), and for no

relationship (H0: $\gamma = 0$, $BF_{0c} = 0.707$, $PostP_0 = .261$). For negative memories, we compared evidence for a positive relationship between episodic detail and corrugator responses (H1: $\gamma > 0$, $BF_{1c} = 0.604$, $PostP_1 = .168$), for a negative relationship (H2: $\gamma < 0$, $BF_{2c} = 1.657$, $PostP_2 = 0.279$), and for no relationship (H0: $\gamma = 0$, $BF_{0c} = 2.476$, $PostP_0 = .553$). The results indicate that more episodic detail is associated with stronger affective responses to positive but not negative memories. Even though this finding was based on few data points from the pilot and needs to be interpreted with caution, it provided preliminary evidence that the ROAM allows to investigate the relationship between episodic detail of autobiographical memories and the psychophysiological expression of affective responses. However, it is important to note that in contrast to the pilot, results from the registered report provided evidence against a relationship of episodic detail and affective responses.

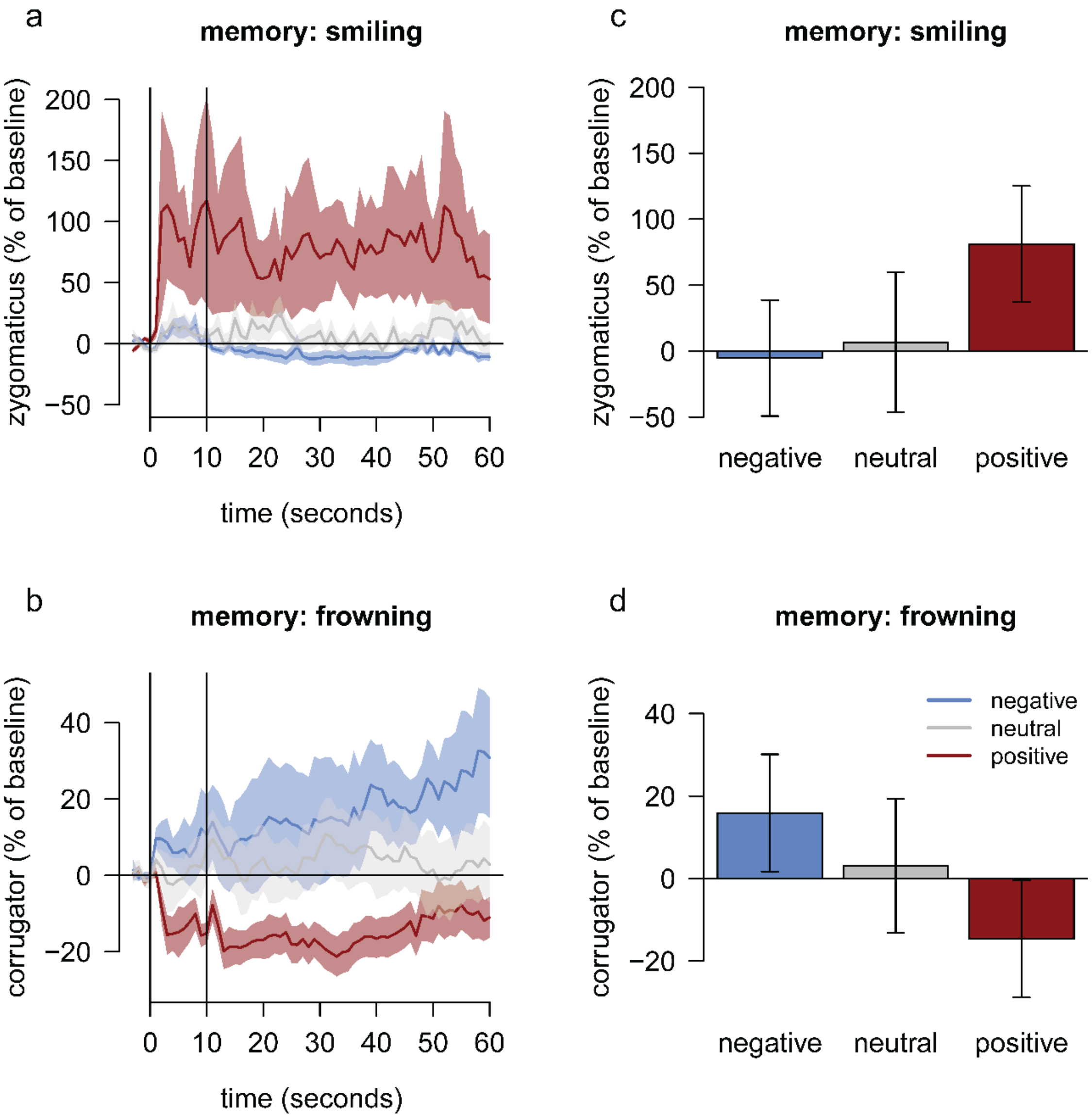

Supplementary Fig. 3 Zygomaticus and corrugator activity during autobiographical memory

recall in the ROAM in the final pilot (*N* = 8 participants). a and **b** present zygomaticus and corrugator activity over time for the positive, negative, and neutral condition, respectively. Muscle activity is presented as percentage change from baseline (4s). The first vertical line indicates the onset of the memory cues (0 s), the second vertical line indicates the offset of the memory cues (10 s). For the remaining 50 seconds participants kept silently reliving the memory. The error bands represent the standard error of the mean. The data was averaged across memories within each participant before calculating the average and standard error for each time point across participants. **c** and **d** represent the estimated average of the zygomaticus and corrugator responses during the recall phase (60 s). The error bars represent credible intervals[95].

Affective expression while watching emotional movie clips. We investigated whether the experience of new positive and negative events (i.e. watching a movie clip) results in enhanced zygomaticus and corrugator activity, respectively (**Supplementary Fig. 4**). We evaluated evidence for the hypotheses that zygomaticus activity is higher while watching positive movie clips compared to neutral movie clips (zygomaticus check 3 – H1, BF_{1c} = 1184.247, $PostP_1 > .988$), that zygomaticus activity is smaller while watching positive movie clips compared to neutral clips (zygomaticus check 3 – H2, $BF_{2c} < 0.001$, $PostP_2 < .001$), and that zygomaticus activity is similar when watching positive movie clips compared to neutral clips (zygomaticus check 3 – H0, BF_{0c} = 0.023, $PostP_0$ = .011). The results provide overwhelming evidence that the zygomaticus is more active when watching positive movies than when watching neutral movies. Further, we evaluated evidence for the hypotheses that corrugator activity is higher while watching negative movie clips compared to neutral movie clips (corrugator check 3 – H1, BF_{1c} = 41.286, $PostP_1$ = .797), that corrugator activity is smaller while watching negative movie clips compared to neutral clips (corrugator check 3 – H2, BF_{2c} = 0.024, $PostP_2$ = .019), and that corrugator activity is similar when watching negative movie clips compared to neutral clips (corrugator check 3 – H0, BF_{0c} = 0.450, $PostP_0$ = .184). The results provide strong evidence that the corrugator is more active when watching negative movies than when watching neutral movies.

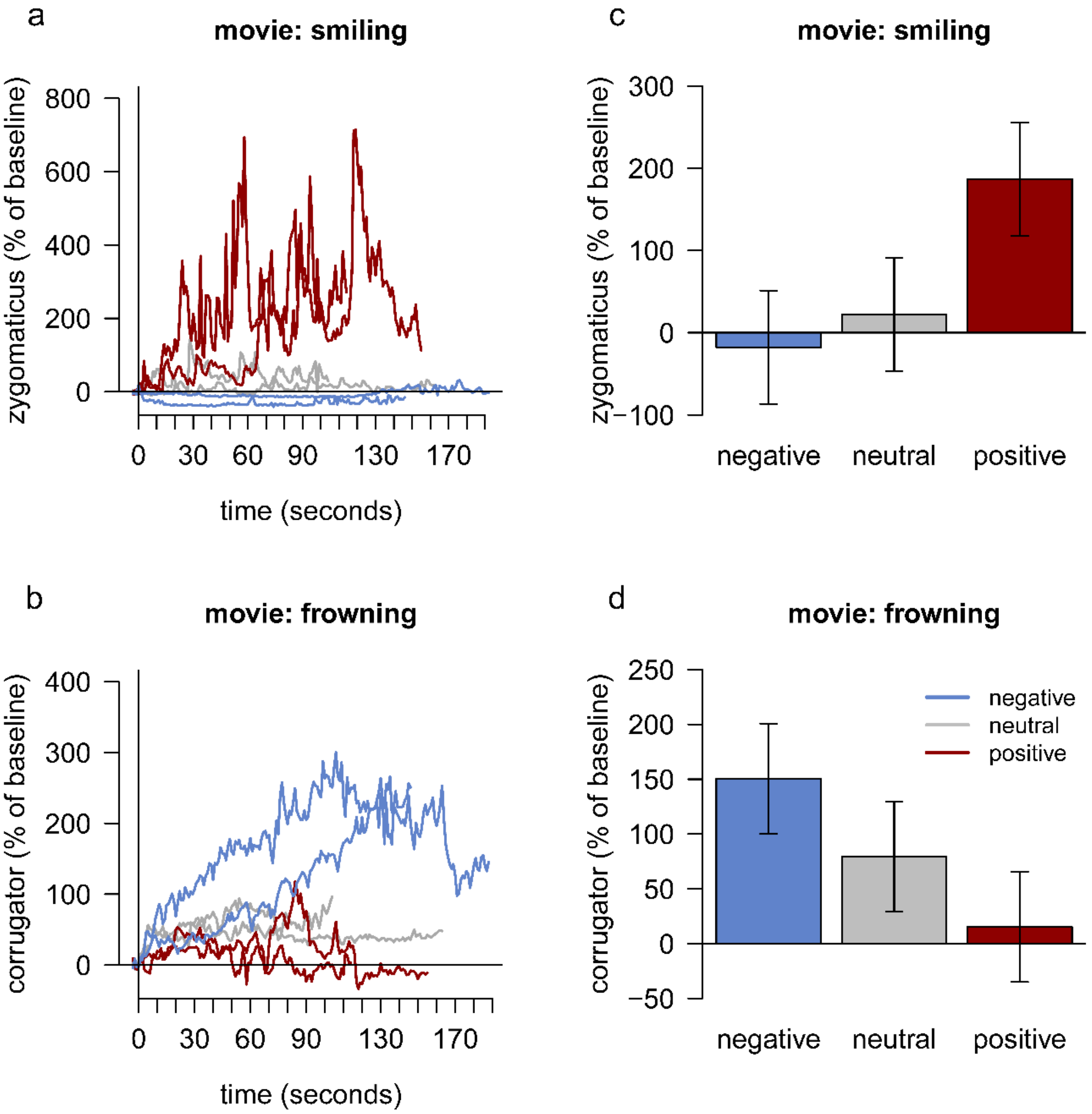

Supplementary Fig. 4 Zygomaticus and corrugator activity while watching movie clips. a and **b** present zygomaticus and corrugator activity over time for movies in the positive, negative, and neutral condition, respectively. Muscle activity is presented as percentage change from baseline (4s). Since the movies have slightly different durations, data was not averaged across movies. Instead, the average timeline across participants is plotted for each movie clip, i.e. two movies per emotion condition. **c** and **d** represent the estimated average (derived from multilevel modelling) of the zygomaticus and corrugator activity while watching movie clips (60 s). The error bars represent credible intervals[96]. The figure represents data from N = 8 participants.

SUPPLEMENTARY NOTE 3:
PILOT SENSITIVITY ANALYSES

Supplementary table 1. Zygomaticus – Manipulation check 1.1.
Do participants without dysphoria smile when remembering positive memories?

	Fraction = 1		Fraction = 2		Fraction = 3	
	BF_{ic}	$PostP_i$	BF_{ic}	$PostP_i$	BF_{ic}	$PostP_i$
H1:	422.414	.957	422.414	.968	422.414	.974
H2:	0.002	.002	0.002	.002	0.002	.002
H0:	0.085	.041	0.060	.029	0.049	.024

H1: Individuals without dysphoria smile more when reliving positive memories than during baseline.
H2: Individuals without dysphoria smile less when reliving positive memories than during baseline.
H0: Individuals without dysphoria smile equally when reliving positive memories and during baseline.

Supplementary table 2. Zygomaticus – Manipulation check 1.2:
Do participants without dysphoria smile more when remembering positive memories compared to neutral memories?

	Fraction = 1		Fraction = 2		Fraction = 3	
	BF_{ic}	$PostP_i$	BF_{ic}	$PostP_i$	BF_{ic}	$PostP_i$
H1:	57.948	.774	57.948	.825	57.948	.850
H2:	0.017	.013	0.017	.014	0.017	.015
H0:	0.541	.213	0.382	.161	0.312	.135

H1: Individuals without dysphoria smile more when reliving positive memories than when remembering neutral memories.
H2: Individuals without dysphoria smile less when reliving positive memories than when remembering neutral memories.
H0: Individuals without dysphoria smile equally when reliving positive memories and neutral memories.

Supplementary table 3. Corrugator – Manipulation check 1.1:
Do participants without dysphoria frown when remembering negative memories?

	Fraction = 1		Fraction = 2		Fraction = 3	
	BF_{ic}	$PostP_i$	BF_{ic}	$PostP_i$	BF_{ic}	$PostP_i$
H1:	44.911	.796	44.911	.842	44.911	.864
H2:	0.022	.018	0.022	.019	0.022	.019
H0:	0.458	.186	0.324	.139	0.117	.117

H1: Individuals without dysphoria frown more when reliving negative memories than during baseline.
H2: Individuals without dysphoria frown less when reliving negative memories than during baseline.
H0: Individuals without dysphoria frown equally when reliving negative memories and during baseline.

Supplementary table 4. Corrugator – Manipulation check 1.2:
Do participants without dysphoria frown more when remembering negative memories compared to neutral memories?

	Fraction = 1		Fraction = 2		Fraction = 3	
	BF_{ic}	$PostP_i$	BF_{ic}	$PostP_i$	BF_{ic}	$PostP_i$
H1:	18.825	.609	18.825	0.680	18.825	0.717
H2:	0.053	.032	0.053	0.036	0.053	0.038
H0:	1.120	.359	0.792	0.284	0.647	0.244

H1: Individuals without dysphoria frown more when reliving negative memories than when remembering neutral memories.
H2: Individuals without dysphoria frown less when reliving negative memories than when remembering neutral memories.
H0: Individuals without dysphoria frown equally when reliving negative memories and neutral memories.

Supplementary table 5. Zygomaticus – Test 3.1A:
Does the amount of retrieved episodic detail predict affective responses to a positive autobiographical memory among individuals without dysphoria?

	Fraction = 1		Fraction = 2		Fraction = 3	
	BF_{ic}	$PostP_i$	BF_{ic}	$PostP_i$	BF_{ic}	$PostP_i$
H1:	18.209	.632	18.209	.700	18.209	.736
H2:	0.055	.035	0.055	.038	0.055	.040
H0:	1.00	.333	0.707	.261	0.577	.224

H1: Within individuals, more episodic detail during memory retrieval predicts stronger affective responses to positive memories.
H2: Within individuals, more episodic detail during memory retrieval predicts weaker affective responses to positive memories.
H0: Within individuals, episodic detail does not predict affective responses to positive memories.

Supplementary table 6. Corrugator – Test 3.1A:
Does the amount of retrieved episodic detail predict affective responses to a negative autobiographical memory among individuals without dysphoria?

	Fraction = 1		Fraction = 2		Fraction = 3	
	BF_{ic}	$PostP_i$	BF_{ic}	$PostP_i$	BF_{ic}	$PostP_i$
H1:	0.604	.137	0.604	0.168	0.604	.187
H2:	1.657	.227	1.657	0.279	1.657	.310
H0:	3.502	.636	2.476	0.553	2.022	.503

H1: Within individuals, more episodic detail during memory retrieval predicts stronger affective responses to negative memories.
H2: Within individuals, more episodic detail during memory retrieval predicts weaker affective responses to negative memories.
H0: Within individuals, episodic detail does not predict affective responses to negative memories.

SUPPLEMENTARY NOTE 4:
PARTICIPANT SCREENING AND INCLUSION

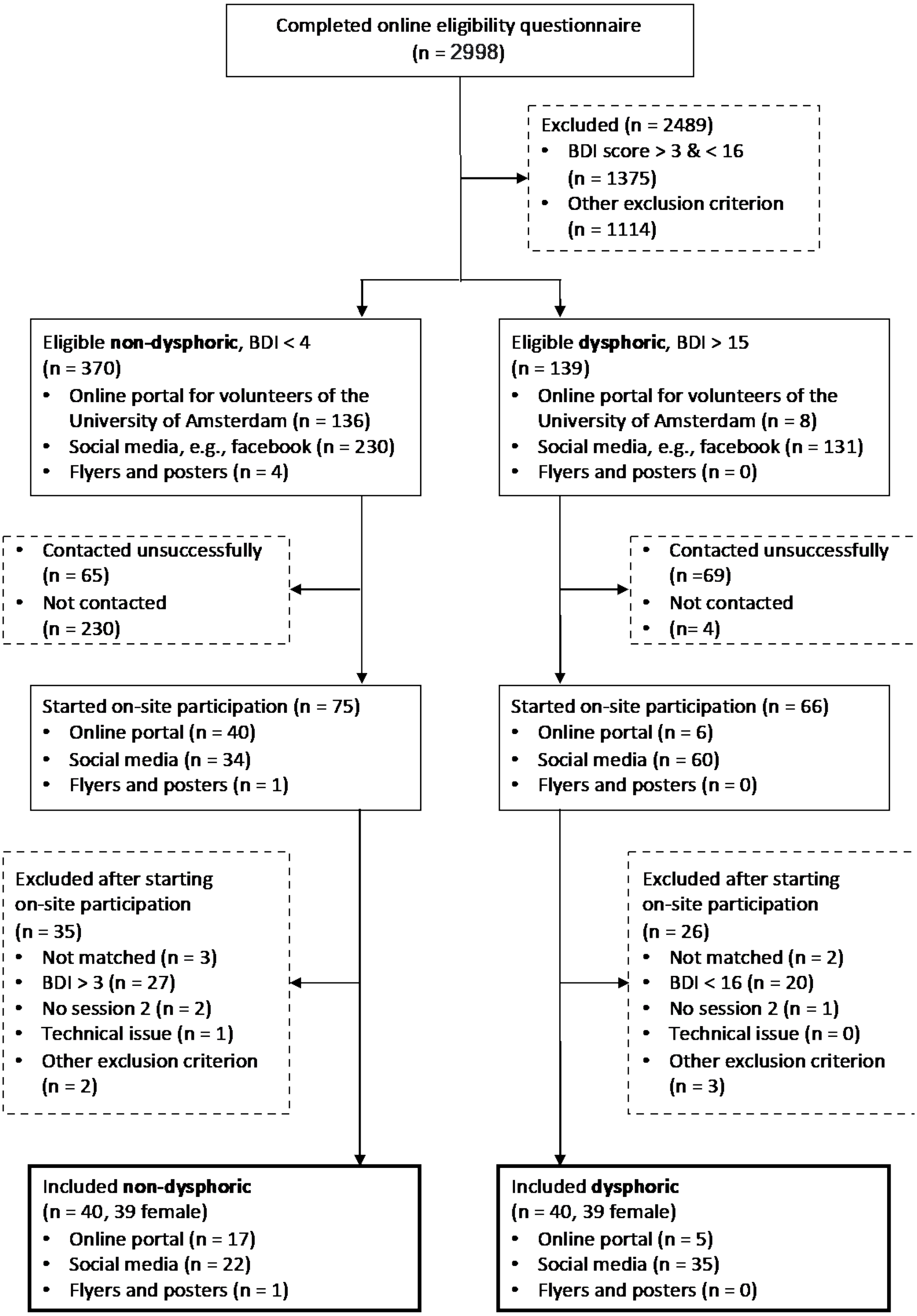

Supplementary Fig. 5 Overview of the screening and inclusion of participants.

SUPPLEMENTARY NOTE 5: BAYESIAN UPDATING

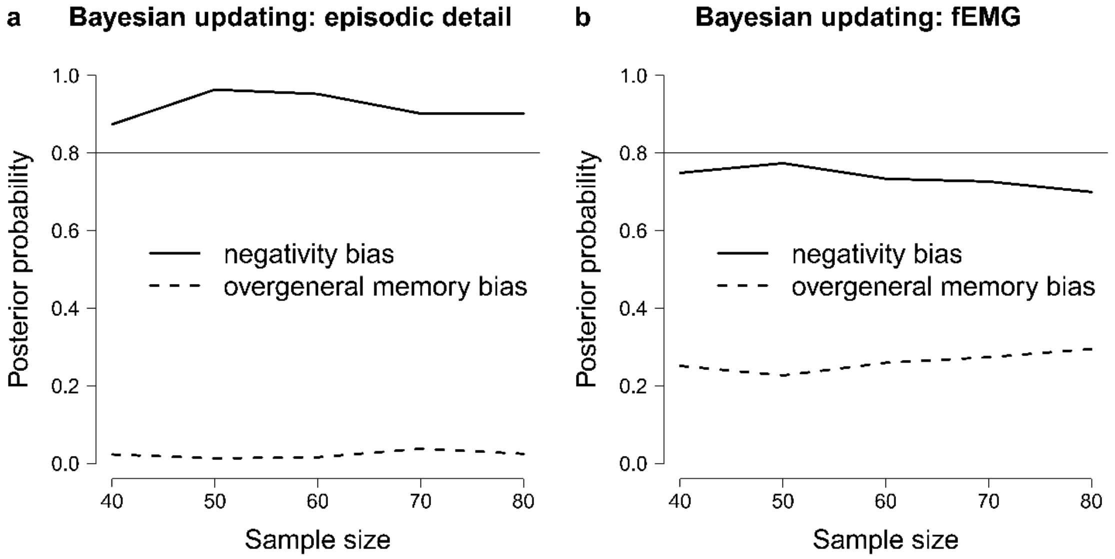

Supplementary Fig. 6 Bayesian Updating. Evidence with increasing sample size from $N = 40$ to $N = 80$ in steps of ten (five per group) for the preregistered analyses of episodic detail (a) and fEMG responses to memories (b). Evidence for the null and for the fail-safe hypothesis is not presented because they overlap and approach zero. The horizontal line at the posterior probability PostP = .8 indicates the stopping criterion.

SUPPLEMENTARY NOTE 6:
COMPLEMENTARY BOXPLOTS

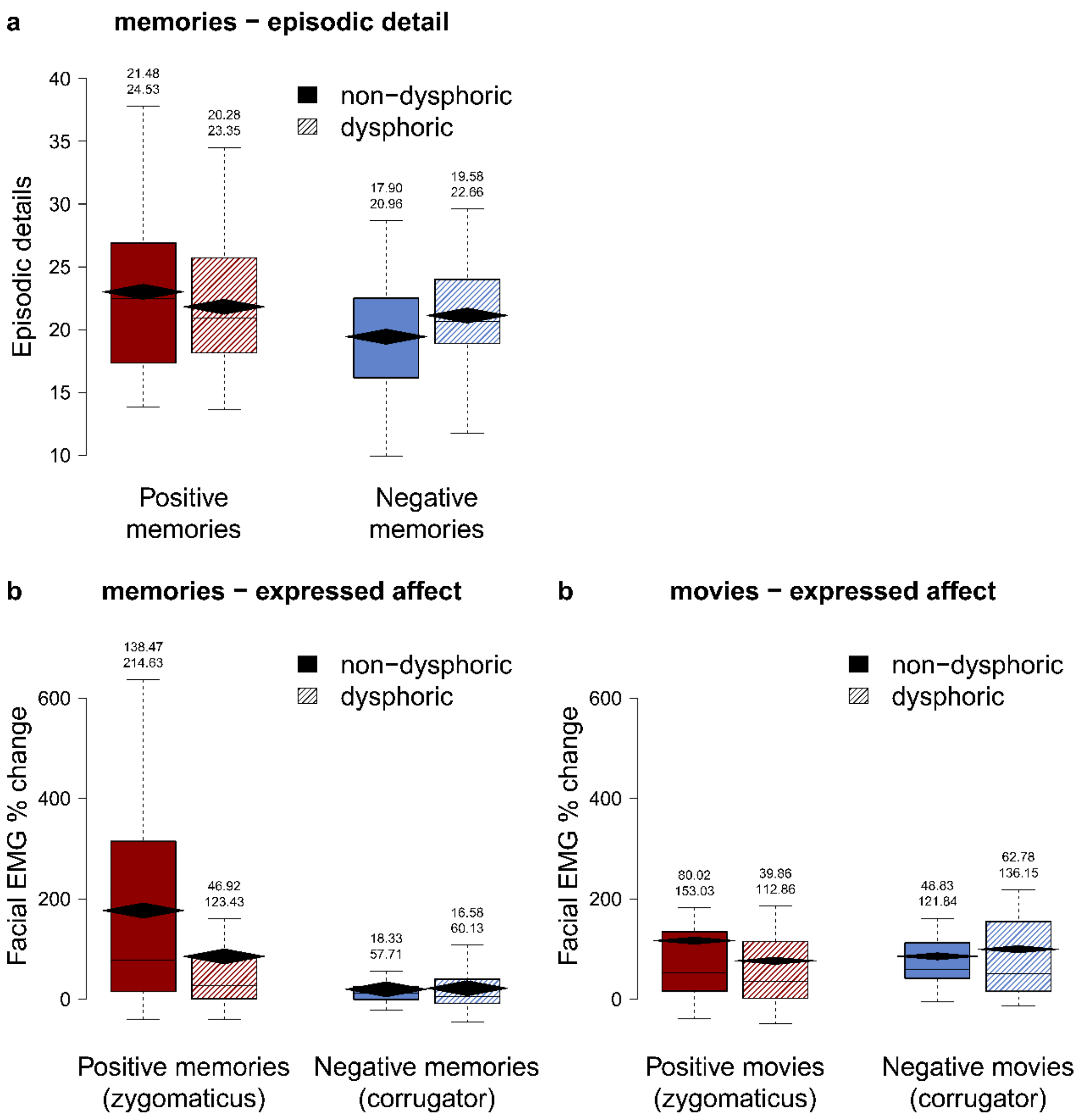

Supplementary Fig. 7 The Re-experience Of Autobiographical Memories (ROAM) in individuals with and without dysphoria. **a** depicts the number of retrieved episodic details when reliving emotional autobiographical memories. **b** and **c** depict the expressed affect while remembering emotional autobiographical memories and while watching emotional movie clips, respectively. For the boxplots, the data were first averaged across memories within condition and participant. The bounds of the boxes depict the interquartile range between the 25th and 75th percentile. The minima and maxima represent 1.5 times the interquartile range below and above the interquartile range, respectively. The horizontal bars indicate the median. The horizontal diamond shapes indicate the estimated means from the multilevel models used to evaluate the confirmatory hypotheses. The two numbers above each box represent the lower and upper bound of the Bayesian credible interval. The figure presents data from 779 memories (a), 781 memories (b), and 319 movie clips (c) of n = 40 participants with and n = 40

participants without dysphoria. Red elements refer to positive memories or movies, blue elements refer to negative memories or movies.

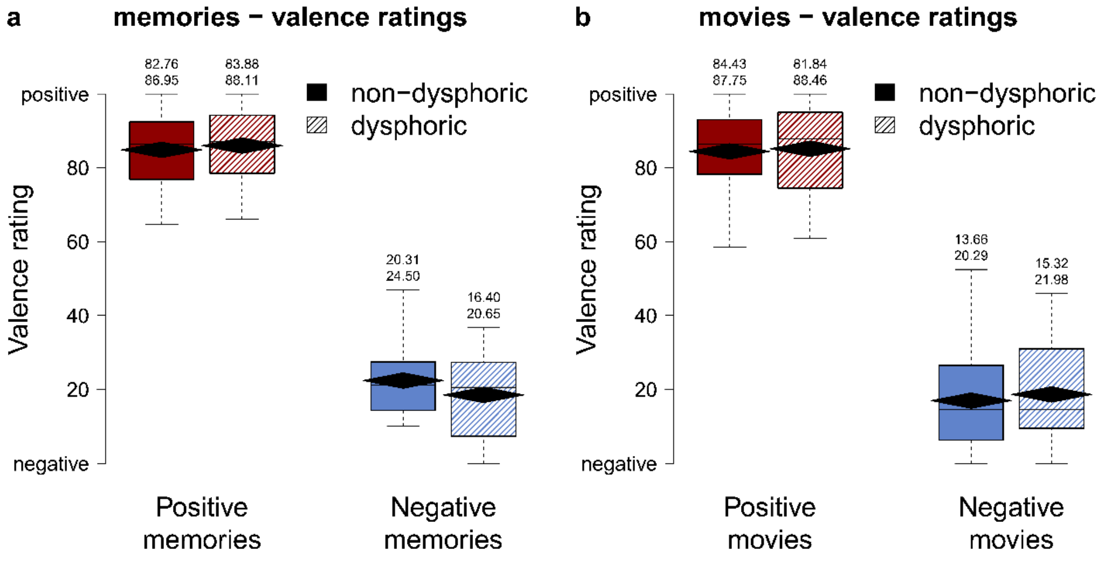

Supplementary Fig. 8 Self-reported feelings of individuals with and without dysphoria when reliving emotional autobiographical memories (a) or watching emotional movie clips (b). For the boxplots, the data were first averaged across memories within condition and participant. The bounds of the boxes depict the interquartile range between the 25th and 75th percentile. The minima and maxima represent 1.5 times the interquartile range below and above the interquartile range, respectively. The horizontal bars indicate the median. The horizontal diamond shapes indicate the estimated means from the multilevel models used to evaluate the confirmatory hypotheses. The two numbers above each box represent the lower and upper bound of the Bayesian credible interval. The figure presents data from 781 memories (a), and 319 movie clips (b) of n = 40 participants with and n = 40 participants without dysphoria. Red elements refer to positive memories or movies, blue elements refer to negative memories or movies.

SUPPLEMENTARY NOTE 7:

FEMG ACTIVITY WHILE WATCHING MOVIE CLIPS

(REGISTERED REPORT DATA)

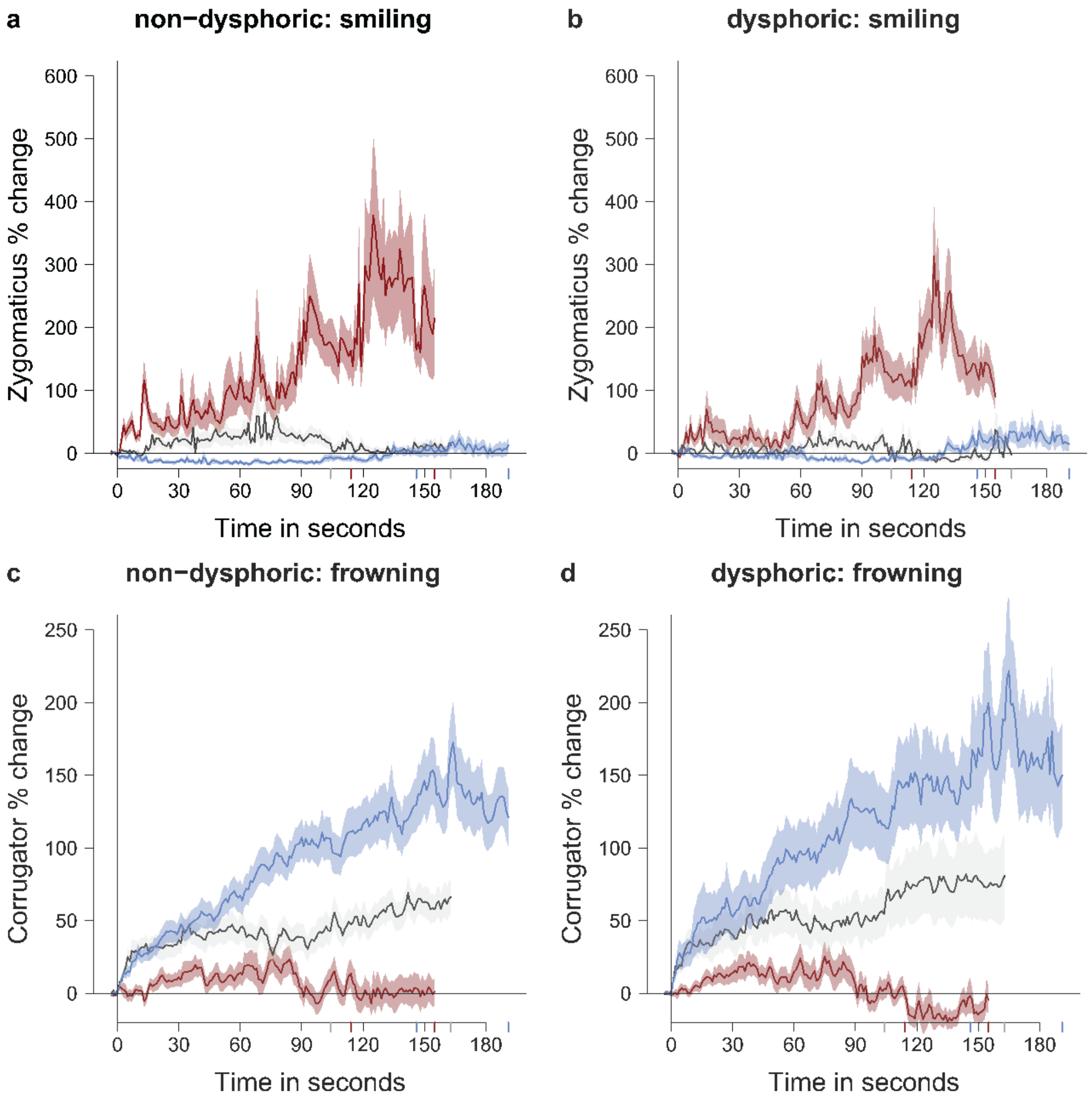

Supplementary Fig. 9 Zygomaticus and corrugator activity of participants with and without dysphoria while watching movie clips. a and **b** present zygomaticus over time in the non-dysphoric and dysphoric group for the positive, negative, and neutral conditions. **c** and **d** present corrugator activity. Muscle activity is presented as percentage change from baseline (4s). The vertical line indicates the onset of the memory cues (0 s). For the remaining 50 seconds participants kept silently reliving the memory. The coloured ticks on the x axis indicate the end of specific movie clips because movies have slightly different durations. The error bands represent the standard error of the mean. The data was averaged across memories within each participant before calculating the average and standard error for each time point across participants.

SUPPLEMENTARY NOTE 8:
RESULTS FROM BAYESIAN INFORMATIVE HYPOTHESIS TESTING INCLUDING SENSITIVITY ANALYSES

Supplementary Table 7. Sensitivity analyses for Bayesian data quality checks for the zygomaticus (**a**) and corrugator (**b**). Since **a** was conducted on the fEMG data before standardization, we conducted an additional quality check in which we tested whether the standardized fEMG response during memory recall was different from 0 (**c**).

a

**zygomaticus**			**non-dysphoric**		**dysphoric**	
		fraction	BF_{iC}	$PostP_i$	BF_{iC}	$PostP_i$
recall	recall > baseline	1	$22.861*10^{12}$	> .999	3796.823	0.988
vs		2	$22.861*10^{12}$	> .999	3796.823	0.991
baseline		3	$22.861*10^{12}$	> .999	3796.823	0.993
	recall < baseline	1	0.000	< .001	0.000	< .001
		2	0.000	< .001	0.000	< .001
		3	0.000	< .001	0.000	< .001
	recall = baseline	1	0.000	< .001	0.024	0.012
		2	0.000	< .001	0.017	0.008
		3	0.000	< .001	0.014	0.007
positive	positive > neutral	1	$41.544*10^{9}$	> .999	9868.495	0.995
vs		2	$41.544*10^{9}$	> .999	9868.495	0.996
neutral		3	$41.544*10^{9}$	> .999	9868.495	0.997
	positive < neutral	1	0.000	< .001	0.000	< .001
		2	0.000	< .001	0.000	< .001
		3	0.000	< .001	0.000	< .001
	positive = neutral	1	0.000	< .001	0.010	.005
		2	0.000	< .001	0.007	.003
		3	0.000	< .001	0.006	.003

b

**corrugator**		fraction	**non-dysphoric** BF_{iC}	$PostP_i$	**dysphoric** BF_{iC}	$PostP_i$
recall vs baseline	recall > baseline	1	67.850	.737	16.654	.457
		2	67.850	.796	16.654	.538
		3	67.850	.825	16.654	.584
	recall < baseline	1	0.015	.011	0.060	.027
		2	0.015	.012	0.060	.032
		3	0.015	.012	0.060	.035
	recall = baseline	1	0.673	.252	2.132	.516
		2	0.476	.192	1.508	.430
		3	0.388	.163	1.231	.381
negative vs neutral	negative > neutral	1	$12.775*10^4$	> .999	46760.609	.999
		2	$12.775*10^4$	> .999	46760.609	.999
		3	$12.775*10^4$	> .999	46760.609	.999
	negative < neutral	1	0.000	< .001	0.000	< .001
		2	0.000	< .001	0.000	< .001
		3	0.000	< .001	0.000	< .001
	negative = neutral	1	0.001	< .001	0.001	.001
		2	0.001	< .001	0.001	.001
		3	0.001	< .001	0.001	.001

c

**exploratory data quality checks**			**non-dysphoric**		**dysphoric**	
		fraction	BF_{iC}	$PostP_i$	BF_{iC}	$PostP_i$
zygo-	recall > 0	1	$22.919*10^{12}$	> .999	1039849	> .999
maticus		2	$22.919*10^{12}$	> .999	1039849	> .999
		3	$22.919*10^{12}$	> .999	1039849	> .999
positive	recall < 0	1	0.000	< .001	0.000	< .001
recall		2	0.000	< .001	0.000	< .001
vs 0		3	0.000	< .001	0.000	< .001
	recall = 0	1	0.000	< .001	0.000	< .001
		2	0.000	< .001	0.000	< .001
		3	0.000	< .001	0.000	< .001
corrugator	recall > 0	1	$48.954*10^{7}$	> .999	$2.181*10^{6}$	> .999
		2	$48.954*10^{7}$	> .999	$2.181*10^{6}$	> .999
negative		3	$48.954*10^{7}$	> .999	$2.181*10^{6}$	> .999
recall	recall < 0	1	0.000	< .001	0.000	< .001
vs 0		2	0.000	< .001	0.000	< .001
		3	0.000	< .001	0.000	< .001
	recall = 0	1	0.000	< .001	0.000	< .001
		2	0.000	< .001	0.000	< .001
		3	0.000	< .001	0.000	< .001

Supplementary Table 8. Sensitivity analyses for the analyses of affective responses measured with fEMG. **a** presents results from the primary preregistered analysis of fEMG responses to memories. **b** presents results from an exploratory analysis including a positive attenuation hypothesis. **c** presents the preregistered analysis of fEMG responses to movies. **d** presents results from an exploratory analysis of responses to movies including a positive attenuation hypothesis.

a

**fEMG**	fraction	BF_{iC}	$PostP_i$
overgeneral memory bias	1	1.650	.292
	2	1.822	.295
	3	1.745	.283
negativity bias	1	5.642	.691
	2	6.061	.700
	3	6.391	.713
null hypothesis	1	0.054	.012
	2	0.027	.006
	3	0.018	.004
fail-safe	1	-	.005
	2	-	< .001
	3	-	< .001

b

**fEMG - exploratory**	fraction	BF_{iC}	$PostP_i$
negativity bias	1	5.947	.121
	2	5.895	.162
	3	6.182	.197
positive attenuation	1	24.113	.879
	2	17.050	.838
	3	13.922	.803

c

**fEMG – movies**	fraction	BF_{iC}	$PostP_i$
negativity bias	1	7.164	.878
	2	7.236	.879
	3	7.222	.878
fail-safe	1	-	.122
	2	-	.121
	3	-	.122

d

**fEMG – movies**	fraction	BF_{iC}	$PostP_i$
negativity bias	1	7.241	.153
	2	7.201	.201
	3	7.207	.236
positive attenuation	1	17.180	.826
	2	12.148	.771
	3	9.919	.732
fail-safe	1	-	.021
	2	-	.028
	3	-	.033

Supplementary Table 9. Sensitivity analyses for the analyses of affective valence measured self-report. **a** presents results from the primary preregistered analysis of the memory data. **b** presents the preregistered analysis of the movie data.

a

**valence**	fraction	BF_{iC}	$PostP_i$
overgeneral memory bias	1	0.805	.083
	2	0.803	.134
	3	0.811	.520
negativity bias	1	0.003	< .001
	2	0.003	.001
	3	0.003	.001
null hypothesis	1	7.814	.765
	2	3.907	.619
	3	2.605	.520
fail-safe	1	-	.151
	2	-	.246
	3	-	.309

b

**valence – movies**	fraction	BF_{iC}	$PostP_i$
null hypothesis	1	114.357	.991
	2	57.178	.983
	3	114.357	.991
fail-safe (complement)	1	-	.009
	2	-	.017
	3	-	.009

Supplementary Table 10. Sensitivity analyses for the analyses of episodic detail.

**episodic detail**	fraction	BF_{iC}	$PostP_i$
overgeneral memory bias	1	0.118	.023
	2	0.113	.025
	3	0.109	.022
negativity bias	1	28.767	.882
	2	26.219	.902
	3	24.688	.951
null hypothesis	1	0.438	.059
	2	0.219	.034
	3	0.146	.022
fail-safe	1	-	.036
	2	-	.042
	3	-	.046

Supplementary Table 11. Sensitivity analyses for the relationship of fEMG responses to memories and episodic detail. **a** presents the results regarding the relationship between zygomaticus responses to positive memories and episodic detail for participants with and without dysphoria, **b** presents results regarding group differences in this relationship. **c** and **d** present the same for the relationship of corrugator responses to negative memories and episodic detail. dys: dysphoric; non-dys: non-dysphoric.

a

**positive memories - zygomaticus**			**non-dysphoric**		**dysphoric**	
		fraction	BF_{iC}	$PostP_i$	BF_{iC}	$PostP_i$
within-participant relationship of affect & detail	$\gamma > 0$	1	0.727	.059	0.340	.042
		2	0.727	.079	0.340	.056
		3	0.727	.093	0.340	.065
	$\gamma < 0$	1	1.376	.081	2.939	.124
		2	1.376	.108	2.939	.164
		3	1.376	.127	2.939	.191
	$\gamma = 0$	1	12.281	.860	10.060	.834
		2	8.684	.813	7.114	.781
		3	7.090	780	5.808	.744
between-participant relationship of affect & detail	$\beta > 0$	1	8.511	.232	3.811	.144
		2	8.511	.296	3.811	.189
		3	8.511	.338	3.811	.220
	$\beta > 0$	1	0.117	.027	0.262	.038
		2	0.117	.035	0.262	.050
		3	0.117	.040	0.262	.058
	$\beta = 0$	1	5.716	.741	8.997	.818
		2	4.042	.669	6.361	.761
		3	3.300	.623	5.194	.722

b

group differences - zygomaticus

		fraction	BF_{iC}	$PostP_i$
within-participant relationship of affect & detail	$\gamma_{dys} > \gamma_{non\text{-}dys}$	1	1.737	.092
		2	1.737	.123
		3	1.737	.144
	$\gamma_{dys} < \gamma_{non\text{-}dys}$	1	0.576	.053
		2	0.576	.071
		3	0.576	.083
	$\gamma_{dys} = \gamma_{non\text{-}dys}$	1	11.808	.855
		2	8.350	.807
		3	6.818	.773
between-participant relationship of affect & detail	$\beta_{dys} > \beta_{non\text{-}dys}$	1	1.255	.077
		2	1.255	.103
		3	1.255	.122
	$\beta_{dys} < \beta_{non\text{-}dys}$	1	0.797	.062
		2	0.797	.082
		3	0.797	.097
	$\beta_{dys} = \beta_{non\text{-}dys}$	1	12.402	.861
		2	8.770	.814
		3	7.160	.782

c

**negative memories - corrugator**			**non-dysphoric**		**dysphoric**	
		fraction	BF_{iC}	$PostP_i$	BF_{iC}	$PostP_i$
within-participant relationship of affect & detail	$\gamma > 0$	1	0.141	.030	0.463	.050
		2	0.141	.039	0.463	.066
		3	0.141	.044	0.463	.077
	$\gamma < 0$	1	7.109	.215	2.160	.107
		2	7.109	.275	2.160	.142
		3	7.109	.315	2.160	.166
	$\gamma = 0$	1	6.173	.755	10.776	.843
		2	4.365	.686	7.620	.792
		3	3.564	.641	6.221	.757
between-participant relationship of affect & detail	$\beta > 0$	1	0.285	.040	0.179	.033
		2	0.285	.053	0.179	.043
		3	0.285	.062	0.179	.050
	$\beta < 0$	1	3.512	.142	5.591	.186
		2	3.512	.186	5.591	.241
		3	3.512	.216	5.591	.278
	$\beta = 0$	1	9.002	.818	7.112	.781
		2	6.365	.761	5.029	.715
		3	5.197	.722	4.106	.672

d

group differences - corrugator

		fraction	BF_{iC}	$PostP_i$
within-participant relationship of affect & detail	$\gamma_{dys} > \gamma_{non\text{-}dys}$	1	0.480	.050
		2	0.480	.067
		3	0.480	.078
	$\gamma_{dys} < \gamma_{non\text{-}dys}$	1	2.081	.105
		2	2.081	.139
		3	2.081	.163
	$\gamma_{dys} = \gamma_{non\text{-}dys}$	1	10.890	.845
		2	7.700	.794
		3	6.287	.759
between-participant relationship of affect & detail	$\beta_{dys} > \beta_{non\text{-}dys}$	1	1.538	.089
		2	1.538	.118
		3	1.538	.139
	$\beta_{dys} < \beta_{non\text{-}dys}$	1	0.650	.058
		2	0.650	.077
		3	0.650	.090
	$\beta_{dys} = \beta_{non\text{-}dys}$	1	11.648	.853
		2	8.237	.805
		3	6.725	.771

Supplementary Table 12. Sensitivity analyses for the relationship of valence ratings of memories and episodic detail. **a** presents the results regarding the relationship between valence ratings of positive memories and episodic detail for participants with and without dysphoria, **b** presents results regarding group differences in this relationship. **c** and **d** present the same for valence ratings of negative memories and episodic detail. dys: dysphoric; non-dys: non-dysphoric.

a

**positive memories – valence**			**non-dysphoric**		**dysphoric**	
		fraction	BF_{iC}	$PostP_i$	BF_{iC}	$PostP_i$
within-participant relationship of affect & detail	$\gamma > 0$	1	3.810	.140	2130.129	.973
		2	3.810	.185	2130.129	.981
		3	3.810	.215	2130.129	.984
	$\gamma < 0$	1	0.262	.037	0.000	< .001
		2	0.262	.049	0.000	< .001
		3	0.262	.056	0.000	< .001
	$\gamma = 0$	1	9.288	.823	0.054	.026
		2	6.568	.767	0.038	.019
		3	5.362	.728	0.031	.015
between-participant relationship of affect & detail	$\beta > 0$	1	9.644	.244	4.471	.154
		2	9.644	.310	4.471	.202
		3	9.644	.353	4.471	.235
	$\beta > 0$	1	0.104	.025	0.224	.035
		2	0.104	.032	0.224	.045
		3	0.104	.037	0.224	.053
	$\beta = 0$	1	5.435	.731	8.589	.811
		2	3.843	.658	6.074	.752
		3	3.138	.611	4.959	.713

b

group differences - valence

		fraction	BF_{iC}	$PostP_i$
within-participant relationship of affect & detail	$\gamma_{dys} > \gamma_{non\text{-}dys}$	1	0.034	.015
		2	0.034	.018
		3	0.034	.019
	$\gamma_{dys} < \gamma_{non\text{-}dys}$	1	29.689	.444
		2	29.689	.527
		3	29.689	.575
	$\gamma_{dys} = \gamma_{non\text{-}dys}$	1	2.362	.541
		2	1.670	.455
		3	1.364	.405
between-participant relationship of affect & detail	$\beta_{dys} > \beta_{non\text{-}dys}$	1	1.194	.073
		2	1.194	.098
		3	1.194	.116
	$\beta_{dys} < \beta_{non\text{-}dys}$	1	0.837	.061
		2	0.837	.082
		3	0.837	.097
	$\beta_{dys} = \beta_{non\text{-}dys}$	1	12.854	.865
		2	9.089	.820
		3	7.421	.788

c

**negative memories - valence**			**non-dysphoric**		**dysphoric**	
		fraction	BF_{iC}	$PostP_i$	BF_{iC}	$PostP_i$
within-participant relationship of affect & detail	$\gamma > 0$	1	0.209	.032	0.071	.021
		2	0.209	.042	0.071	.026
		3	0.209	.049	0.071	.029
	$\gamma < 0$	1	4.778	.153	13.992	.289
		2	4.778	.201	13.992	.362
		3	4.778	.233	13.992	.408
	$\gamma = 0$	1	8.835	.815	4.465	.691
		2	6.247	.757	3.157	.612
		3	5.101	.718	2.578	.563
between-participant relationship of affect & detail	$\beta > 0$	1	0.692	.053	0.137	.027
		2	0.692	.071	0.137	.035
		3	0.692	.084	0.137	.040
	$\beta < 0$	1	1.445	.077	7.297	.197
		2	1.445	.103	7.297	.255
		3	1.445	.121	7.297	.293
	$\beta = 0$	1	13.411	.870	6.926	.776
		2	9.483	.826	4.897	.710
		3	7.743	.795	3.999	.667

d

group differences - valence

		fraction	BF_{iC}	$PostP_i$
within-participant relationship of affect & detail	$\gamma_{dys} > \gamma_{non\text{-}dys}$	1	2.011	.092
		2	2.011	.123
		3	2.011	.145
	$\gamma_{dys} < \gamma_{non\text{-}dys}$	1	0.497	.046
		2	0.497	.061
		3	0.497	.072
	$\gamma_{dys} = \gamma_{non\text{-}dys}$	1	12.531	.862
		2	8.861	.816
		3	7.235	.783
between-participant relationship of affect & detail	$\beta_{dys} > \beta_{non\text{-}dys}$	1	3.309	.123
		2	3.309	.163
		3	3.309	.190
	$\beta_{dys} < \beta_{non\text{-}dys}$	1	0.302	.037
		2	0.302	.049
		3	0.302	.057
	$\beta_{dys} = \beta_{non\text{-}dys}$	1	10.534	.840
		2	7.448	.788
		3	6.082	.753

Supplementary Table 13. Sensitivity analyses for explorative analyses of zygomaticus and corrugator responses over the course of task.

**fEMG responses**			**non-dysphoric**		**dysphoric**	
		fraction	BF_{iC}	$PostP_i$	BF_{iC}	$PostP_i$
zygomaticus	habituation	1	2.392	.016	0.213	.009
		2	2.578	.063	0.205	.031
		3	2.533	.132	0.210	.059
	null hypothesis	1	147.947	.016	21.418	.946
		2	36.987	.936	5.355	.815
		3	16.439	.125	2.380	.661
	Fail-safe	1	-	.007	-	.044
		2	-	.024	-	.153
		3	-	.050	-	.280
corrugator	habituation	1	0.507	.003	0.043	.008
		2	0.482	.011	0.047	.021
		3	0.500	.025	0.046	.030
	null hypothesis	1	164.817	.991	4.676	.816
		2	41.204	.965	1.169	.526
		3	18.313	.924	0.520	.330
	Fail-safe	1	-	.006	-	.176
		2	-	.024	-	.453
		3	-	.051	-	.640

Supplementary Table 14. Sensitivity analyses for explorative analyses of depressive symptom severity within the dysphoric group as a predictor of episodic detail.

**BDI – episodic detail association**			**dysphoric**	
		fraction	BF_{iC}	$PostP_i$
positive memories	$\beta > 0$	1	1.536	.117
		2	1.536	.153
		3	1.536	.177
	$\beta < 0$	1	0.651	.076
		2	0.651	.099
		3	0.651	.115
	$\beta = 0$	1	8.383	.907
		2	5.928	.748
		3	4.840	.708
negative memories	$\beta > 0$	1	5.050	.202
		2	5.050	.259
		3	5.050	.297
	$\beta < 0$	1	0.198	.040
		2	0.198	.051
		3	0.198	.059
	$\beta = 0$	1	6.273	.758
		2	4.435	.689
		3	3.621	.644

Supplementary Table 15. Sensitivity analyses for explorative analyses of depressive symptom severity within the dysphoric group as a predictor of fEMG responses to emotional memories.

**BDI – fEMG response association**			**dysphoric**	
		fraction	BF_{iC}	$PostP_i$
zygomaticus	$\beta > 0$	1	1.082	.101
		2	1.082	.132
positive		3	1.082	.153
memories	$\beta < 0$	1	0.924	.093
		2	0.924	.122
		3	0.924	.142
	$\beta = 0$	1	8.295	.806
		2	5.866	.746
		3	4.789	.705
corrugator	$\beta > 0$	1	0.218	.048
		2	0.218	.061
negative		3	0.218	.069
memories	$\beta < 0$	1	4.595	.220
		2	4.595	.280
		3	4.595	.318
	$\beta = 0$	1	5.469	.732
		2	3.867	.659
		3	3.157	.612

Supplementary Table 16. Sensitivity analyses for analyses of positive (**a**) and negative (**b**) affect before and after the memory task. Positive and negative affect were assessed with the PANAS.

a

positive affect before and after the memory task

		fraction	BF_{iC}	$PostP_i$
Group differences in affect	dysphoric < non-dysphoric	1	1.488	.142
		2	1.378	.220
		3	1.417	.282
pre & post task	dysphoric = non-dysphoric	1	6.739	.763
		2	3.369	.620
		3	2.246	.519
	fail-safe	1	-	.095
		2	-	.160
		3	-	.199
Group differences in affect change Δ	dysphoric < non-dysphoric	1	0.049	.022
		2	0.049	.026
		3	0.049	.028
	dysphoric > non-dysphoric	1	20.593	.458
		2	20.593	.540
post - pre task		3	20.593	.587
	dysphoric = non-dysphoric	1	2.166	.520
		2	1.532	.434
		3	1.251	.385

b

negative affect before and after the memory task

		fraction	BF_{iC}	$PostP_i$
Group	dysphoric >	1	29.847*107	> .999
differences	non-dysphoric	2	$45.646*10^7$	> .999
in affect		3	$79.150*10^7$	> .999
	dysphoric =	1	0.000	< .001
pre &	non-dysphoric	2	0.000	< .001
post task		3	0.000	< .001
	fail-safe	1	-	< .001
		2	-	< .001
		3	-	< .001
Group	dysphoric >	1	0.441	.062
differences	non-dysphoric	2	0.441	.081
in affect		3	0.441	.797
change Δ	dysphoric <	1	2.267	.094
	non-dysphoric	2	2.267	.184
post -		3	2.267	.213
pre task	dysphoric =	1	7.848	.797
	non-dysphoric	2	5.549	.735
		3	4.531	.694

SUPPLEMENTARY NOTE 9: CHANGES FROM THE INTRODUCTION AND METHODS OF THE STAGE 1 REGISTERED REPORT

- In the methods section of the stage 1 manuscript, we included the erroneous statement:
  "Before each session, we asked about the amount of sleep in the preceding night as well as alcohol and drug use and asked about what films or series they viewed in the night before the session."
  We included such an assessment in other studies on memories of emotional movie clips (https://doi.org/10.31234/osf.io/ukt5x) but we did not include it in this registered that focused on memories of personal events instead of movie clips. Consequently, we deleted this statement.
- In the methods section of the stage 1 manuscript, we specified:
  "This online screening will be conducted by a person who is otherwise not involved in running the experiment, transcribing and coding of the memories. Therefore, the experimenter will be blind regarding the participants' group allocation (dysphoric or non-dysphoric)."
  However, transcribing and coding was time-intense and additional help was required. Therefore, one person who was responsible for the online screening also transcribed and coded several memories (coder initials: RB). For that researcher, all identifying information was removed from the data (e.g. participant number), so that she was blind regarding the participants' group allocation (dysphoric or non-dysphoric). Consequently, we changed the information in the methods section to:
  "This online screening was conducted by a person who was otherwise not involved in running the experiment. The person responsible for the online screening also transcribed and coded several memories but only after all identifying information was removed, so that she was blind regarding the participants' group allocation (dysphoric or non-dysphoric)."
- In the methods section, we stated the following:
  "Specifically, we report self-reported valence and arousal of the memories, the number of retrieved episodic and semantic details, memory age, and memory vividness. For all of these variables, we used BAIT to test whether there are differences between the dysphoric and non-dysphoric group that need to be taken into consideration when interpreting our results."
  However, given the large number of variables and conditions, conducting all of these analyses (at least 39 group comparisons) without strong justification would not be informative because they would likely yield false positive as well as false negative findings. Such potentially spurious findings should better not be considered when interpreting results from the preregistered analyses. Therefore, we did not conduct all possible comparisons. Instead, we conducted a small selection of exploratory analyses with strong justification that were important for the interpretation of our confirmatory findings.
- We removed the information that we might recruit participants through newsletters because we did not use newsletters for recruitment.
- We added a sentence to note that the tentative evidence for an association between episodic detail and fEMG responses to memories in the pilot study was not confirmed in the final registered report data.

- The informed consent for sharing recordings and transcriptions was missing one option (see point 3 below). We added that information: *"Participants further reported on a written informed consent whether we are allowed to share their personal memories 1) without any restrictions on an online platform such as OSF and during presentations, 2) with restrictions on such a platform, 3) only with other researchers upon request, or 4) whether we are not allowed to share their memories."*
- We removed the sentence: *"The mean of the zygomaticus and corrugator activity will represent positive and negative movie emotionality, respectively."* This sentence was accidently not removed during the revisions of the stage 1 protocol when we incorporated feedback from a reviewer to use percentage change for fEMG data instead of means that are not corrected for baseline fEMG activity or difference scores from baseline. We replaced the sentence with: *"The 4 seconds directly preceding each movie onset were used as baseline."*
- We deleted the overgeneral memory bias example for Test 2B to improve readability (given that the data was not in line with an overgeneral memory bias anyway): *"If Test 2A provided evidence for an overgeneral memory bias, we will compare evidence for the hypothesis that individuals with dysphoria show reduced affective responses to negative and positive movies compared to individuals without dysphoria (Test 2B – H1) and evidence for the hypothesis that individuals with dysphoria show any other pattern of affective responses to movies (Test 2B – Hc)."*
- We deleted the references to the design tables from the introduction to improve readability and the structure of the supplemental materials. The design tables are still referenced in the methods section.

SUPPLEMENTARY NOTE 10:

MEMORY SELECTION SHEET – DUTCH ORIGINAL

AUTOBUIOGRAPHICAL MEMORY TASK

PPNR: Date: Version: 1

In deze taak zullen we je vragen om dertien herinneringen te kiezen van gebeurtenissen die je *zelf* hebt meegemaakt. We zullen je vragen om voor iedere herinnering drie cue-woorden op te schrijven die je zullen helpen om de herinnering in de volgende sessie op te halen. Zo'n herinnering kan verwijzen naar iets dat recent of juist langer geleden is gebeurd. Belangrijk is dat de gebeurtenis of het moment van de herinnering *minstens één dag geleden* en *hoogstens vijf jaar geleden* heeft plaatsgevonden. Je zult dus herinneringen opschrijven die minstens één dag oud zijn, maar niet ouder zijn dan vijf jaar.

Specifiek zullen we je vragen om vijf herinneringen te kiezen van gebeurtenissen die je onmiskenbaar als *gelukkig* hebt ervaren en vijf herinneringen van gebeurtenissen die je onmiskenbaar als *verdrietig* hebt ervaren. Dit betekent dat je vijf herinneringen kiest van gebeurtenissen waarin je je gelukkig voelde en dat je vijf herinneringen kiest van gebeurtenissen waarin je je verdrietig voelde. Het gaat dus om gebeurtenissen die voor jou een emotionele betekenis hadden. Bedenk wel dat we je later zullen vragen de herinneringen in iets meer detail te beschrijven. Dit gebeurt anoniem, dus niet van persoon tot persoon. Kies dus alleen herinneringen waarbij je je *comfortabel* voelt de inhoud te delen voor dit onderzoek. Vanzelfsprekend behandelen we alles wat je deelt *vertrouwelijk*. Tot slot vragen we je nog om drie neutrale herinneringen te kiezen. Het is belangrijk dat die neutrale herinneringen niet gekenmerkt worden door een verdrietige of gelukkige emotie, maar dat ze voor jou echt een neutrale waarde hebben.

Verder is het belangrijk dat iedere gelukkige, verdrietige, of neutrale herinnering die je kiest verwijst naar een *specifieke* gebeurtenis. Met specifiek wordt bedoeld dat de herinnering *één bepaalde gebeurtenis* betreft die op een bepaalde dag en plek heeft plaatsgevonden, maar *niet langer dan één dag* heeft geduurd.

Als gelukkige herinnering zou je bijvoorbeeld kunnen kiezen voor *'ik voel me steeds goed op feestjes'*. Deze herinnering is echter niet specifiek, het verwijst niet naar één bepaalde gebeurtenis die op een bepaalde dag plaatsvond. Als je zou kiezen voor *'ik voelde me goed op het laatste feestje bij Veerle'* is dit beter. Dit is een specifieke gebeurtenis. Als herinnering zou je ook kunnen kiezen voor *'vorige zomer voelde ik me goed'*, maar dit verwijst naar een gebeurtenis die langer dan één dag heeft geduurd. Een specifieke gebeurtenis is daarentegen iets dat *één bepaalde keer* als dusdanig is gebeurd en *korter* heeft geduurd dan *één dag*.

Het is de bedoeling dat je voor iedere herinnering *drie cue-woorden* noteert die je kunnen helpen om de herinnering op te halen. Belangrijk is dat de cue-woorden voor jou direct en onmiskenbaar naar deze herinnering verwijzen. Kies dus een woord of een combinatie van woorden die specifiek genoeg zijn. Als cue-woorden voor *'het laatste verjaardagsfeestje bij Veerle'* zou je bijvoorbeeld kunnen

kiezen voor de specifieke cue-woorden *'feestje Veerle'*, in plaats van het bredere cue-woord *'verjaardag'*.

Neem zolang de tijd als nodig is om je herinneringen uit te kiezen en de cue-woorden te noteren. Belangrijk is dat je ***dertien verschillende*** herinneringen of gebeurtenissen uitkiest. Het is dus niet de bedoeling dat je tweemaal cue-woorden opschrijft voor exact eenzelfde gebeurtenis of herinnering. Geef ook aan hoe lang geleden het ongeveer is dat de gebeurtenis van de herinnering plaatsvond (in maanden). Als de herinnering minder dan een maand oud is, geef dan aan hoe lang geleden de gebeurtenis van de herinnering plaatsvond in weken.

GELUKKIGE HERINNERINGEN

Kies vijf herinneringen van gebeurtenissen die je onmiskenbaar als ***gelukkig*** ervaart. Noteer voor iedere herinnering ***drie cue-woorden*** die je kunnen helpen om de herinnering op te halen. Belangrijk is dat de gebeurtenis ***minstens één dag geleden*** en ***hoogstens vijf jaar geleden*** heeft plaatsgevonden.

Gelukkige herinnering 1 Hoe lang geleden: _____ maanden, _____ weken

Cue-woorden: ____

Gelukkige herinnering 2 Hoe lang geleden: _____ maanden, _____ weken

Cue-woorden: ____

Gelukkige herinnering 3 Hoe lang geleden: _____ maanden, _____ weken

Cue-woorden: ____

Gelukkige herinnering 4 Hoe lang geleden: _____ maanden, _____ weken

Cue-woorden: ____

Gelukkige herinnering 5 Hoe lang geleden: _____ maanden, _____ weken

Cue-woorden: ____

VERDRIETIGE HERINNERINGEN

Kies vijf herinneringen van gebeurtenissen die je onmiskenbaar als ***verdrietig*** ervaart. Noteer voor iedere herinnering ***drie cue-woorden*** die je kunnen helpen om de herinnering op te halen. Belangrijk is dat de gebeurtenis ***minstens één dag geleden*** en ***hoogstens vijf jaar geleden*** heeft plaatsgevonden.

Verdrietige herinnering 1 Hoe lang geleden: _____ maanden, _____ weken

Cue-woorden: ____

Verdrietige herinnering 2 Hoe lang geleden: _____ maanden, _____ weken

Cue-woorden: ____

Verdrietige herinnering 3 Hoe lang geleden: _____ maanden, _____ weken

Cue-woorden: ____

Verdrietige herinnering 4 Hoe lang geleden: _____ maanden, _____ weken

Cue-woorden: ____

Verdrietige herinnering 5 Hoe lang geleden: _____ maanden, _____ weken

Cue-woorden: ____

NEUTRALE HERINNERINGEN

Kies drie herinneringen van gebeurtenissen die je als ***neutraal*** ervaart. Noteer voor iedere herinnering ***drie cue-woorden*** die je kunnen helpen om de herinnering op te halen. Belangrijk is dat de gebeurtenis ***minstens één dag geleden*** en ***hoogstens vijf jaar geleden*** heeft plaatsgevonden.

Neutrale herinnering 1 Hoe lang geleden: _____ maanden, _____ weken

Cue-woorden: ____

Neutrale herinnering 2 Hoe lang geleden: _____ maanden, _____ weken

Cue-woorden: ____

Neutrale herinnering 3 Hoe lang geleden: _____ maanden, _____ weken

Cue-woorden: ____

MEMORY SELECTION SHEET – ENGLISH TRANSLATION

AUTOBIORAFISCHE HERRINERINGENTAAK

PPNR: Datum: Version: 1

In this task, we will ask you to choose thirteen memories of events you have experienced **yourself**. We will ask you to write down three cue words for each memory that will help you to recall the memory in the next session. Such a memory can refer to something that happened recently or a long time ago. It is important that the event or moment of the memory occurred **at least one day ago** and **at most five years ago**. Hence, you will write down memories that are at least one day old, but not more than five years old.

Specifically, we will ask you to choose five memories of events that you clearly experienced as **happy** and five memories of events that you clearly experienced as **sad**. This means that you choose five memories of events during which you felt happy and five memories of events during which you felt sad. Therefore, the memories should have had emotional significance for you. Keep in mind though, that we will ask you to describe the memories more detail later. This will be done anonymously, so not from person to person. Please choose only memories that you feel **comfortable** sharing for this study. Of course, we will treat everything you share **confidentially**. Finally, we ask you to choose three neutral memories. It is important that these neutral memories are not characterized by a sad or happy emotion, but that they really have a neutral value for you.

Moreover, it is important that each happy, sad, or neutral memory that you choose refers to a **specific** event. Specific means that the memory refers to **one particular event** that took place on one day and in one place, and did **not last longer than one day**.

For a happy memory, you might for example choose "*I always feel good at parties*". However, this memory is not specific, it does not refer to one particular event that happened on a particular day. If you chose "*I felt good at the last party at Veerle's place*", this is better. This is a specific event. You could also choose "*last summer I felt good*", but this refers to an event that lasted longer than one day. In contrast, a specific event is something that **happened once** and lasted **for less than one day**.

The idea is to write down **three cue words** for each memory that can help you to recall the memory. It is important that the cue words refer directly and unmistakably to this memory for you. So choose a word or a combination of words that are specific enough. For example, as cue words for "*the last party at Veerle's place*," you might choose the specific cue words "party Veerle," rather than the broader cue word "birthday".

Take as much time as you need to choose your memories and write down the cue words. It is important that you choose **thirteen different** memories or events. That means, you should not write down cue words twice for the exact same event or memory.
Also indicate approximately how long ago the event of the memory took place (in months). If the memory is less than a month old, indicate how long ago the event of the memory occurred in weeks.

HAPPY MEMORIES

Choose five memories of events that you unmistakably experience as **happy**. For each memory, write down **three cue words** that can help you to recall the memory. It is important that the event happened **at least one day ago** and **at most five years ago**.

Happy memory 1 How long ago: _____ months, _____ weeks

Cue-words: ____

Happy memory 2 How long ago: _____ months, _____ weeks

Cue-words: ____

Happy memory 3 How long ago: _____ months, _____ weeks

Cue-words: ____

Happy memory 4 How long ago: _____ months, _____ weeks

Cue-words: ____

Happy memory 5 How long ago: _____ months, _____ weeks

Cue-words: ____

SAD MEMORIES

Choose five memories of events that you unmistakably experience as **sad**. For each memory, write down **three cue words** that can help you to recall the memory. It is important that the event happened **at least one day ago** and **at most five years ago**.

Sad memory 1 How long ago: _____ months, _____ weeks

Cue-words: ____

Sad memory 2 How long ago: _____ months, _____ weeks

Cue-words: ____

Sad memory 3 How long ago: _____ months, _____ weeks

Cue-words: ____

Sad memory 4 How long ago: _____ months, _____ weeks

Cue-words: ____

Sad memory 5 How long ago: _____ months, _____ weeks

Cue-words: ____

NEUTRAL MEMORIES

Choose five memories of events that you unmistakably experience as **neutral**. For each memory, write down **three cue words** that can help you to recall the memory. It is important that the event happened **at least one day ago** and **at most five years ago**.

Neutral memory 1 **How long ago: _____ months, _____ weeks**

Cue-words: ____

Neutral memory 2 **How long ago: _____ months, _____ weeks**

Cue-words: ____

Neutral memory 3 **How long ago: _____ months, _____ weeks**

Cue-words: ____

SUPPLEMENTARY NOTE 11:
MOVIE CLIP DESCRIPTIONS

Supplementary table 17. Movie clip desriptions.

condition	movie	scene description	approx. time in movie	duration
positive	**Marley and Me**<br><br>Rosenfelt, K. (producer), & Frankel, D. (director). (2008).	Labrador Marley and John are at the beach. John is talking to Marley about life. He lets Marley off the leash against orders to play in the water. Marley enjoys his freedom and other dog owners release their dogs as well. They have a great time.	1:12:15	115 seconds
	**Untouchable**<br><br>Duval, N., (producer), & Nakache, O. Toledano, É . (directors). (2011).	Philippe takes Driss paragliding. Driss refuses at first, but joins Philippe reluctantly. In the end, he is thrilled and enjoys it.	1:20:25	156 seconds
negative	**Secret in Their Eyes**<br><br>Jackson, M., (producer), & Ray, B. (director). (2015).	Jess, Ray, Bumpy and their colleague arrive at a crime scene in an underground carpark. A policeman shows Ray the corpse in a dumpster. Ray slowly walks to Jess, and tells her that it is her daughter. She runs to the dumpster, climbs in, holds her daughter and is crying in despair.	53:45	192 seconds
	**The Impossible**<br><br>Augustin, Á., (producer), & Bayona, J. A. (director). (2012).	A man calls home after a tsunami, saying that he cannot find his wife or children.	1:01:37	147 seconds
neutral	**Dead Poet Society**	Teachers and students are in the dining hall for lunch. A teacher, Mr. McAllister, talks	26:50	105 seconds

	Haft, S. (producer), & Weir, P. (director). (1989)	to Mr. Keating about his teaching style. Neil found Mr. Keating´s old yearbook and shows it to Richard and other students.		
	**Big Night**<br><br>Kirkpatrick, D. (producer), & Scott, C., Tucci, S. (directors). (1996).	Secundo and Primo are cooking in a big kitchen. Their waiter, Cristiano, joins and wants to get water from the sink, but it is broken. Secundo leaves the kitchen, prepares the restaurant for the guests and opens the restaurant´s doors.	02:00	164 seconds

SUPPLEMENTARY NOTE 12:
BLINDING

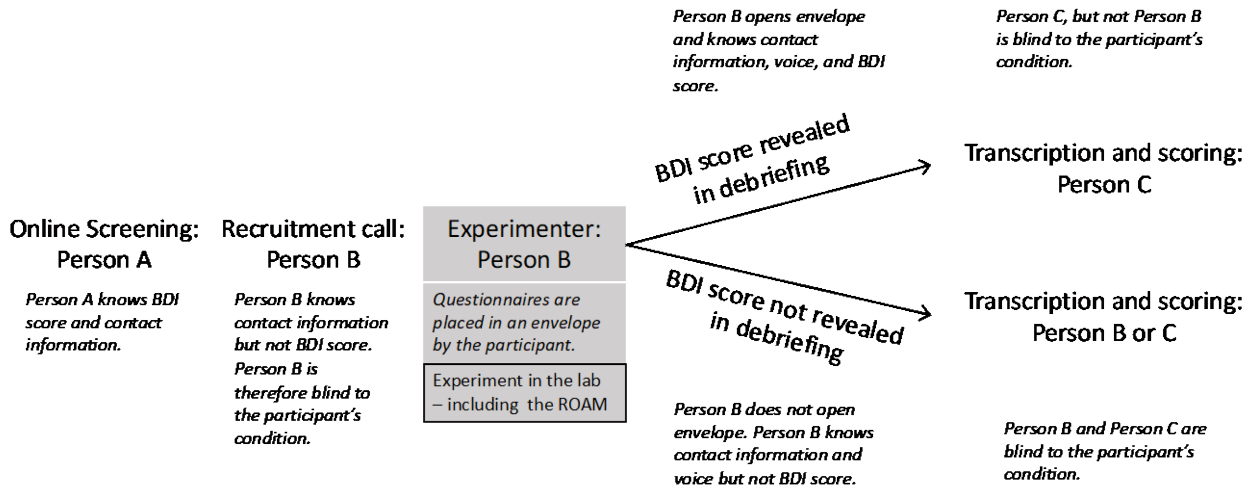

Supplementary Fig. 10 Overview of the procedure to ensure blinding. One person responsible for the online screening also transcribed and coded several memories. For that person (initials: RB), the memories were anonymized prior to transcription and coding.

SUPPLEMENTARY NOTE 13:
DESIGN TABLES

OVERVIEW OF DESIGN TABLES

- **Design Table 1: Manipulation checks**
  - Zygomaticus activity during positive memory recall
    - Manipulation check 1.1: Do participants without dysphoria smile when remembering positive memories?
    - Manipulation check 1.2: Do participants without dysphoria smile more when remembering positive memories compared to neutral memories?
  - Corrugator activity during negative memory recall
    - Manipulation check 2.1: Do participants without dysphoria frown when remembering negative memories?
    - Manipulation check 2.2: Do participants without dysphoria frown more when remembering negative memories compared to neutral memories?
- **Design Table 2: Confirmatory analyses – Test 1 – episodic detail**
  - Which theory best explains episodic memory distortions in dysphoria?
- **Design Table 3: Confirmatory analyses – Test 2A and 2B – affective responses**
  - Test 2A: Which theory best explains affective memory distortions in dysphoria?
  - Test 2B: Domain-specificity of affective responses: Are the distorted affective responses to autobiographical memories specific to memories or already present during the encoding of events?
- **Design Table 4: Confirmatory analyses – Test 3 – relationship of episodic detail and affective responses**
  - Positive memories
    - Test 3.1A: Does the amount of retrieved episodic detail predict affective responses to positive memories among individuals with and without dysphoria?
    - Test 3.1B: Does the within-participant relationship between episodic detail and affective responses differ between individuals with and without dysphoria?
  - Negative memories
    - Test 3.2A: Does the amount of retrieved episodic detail predict affective responses to negative memories among individuals with and without dysphoria?
    - Test 3.2B: Does the within-participant relationship between episodic detail and affective responses differ between individuals with and without dysphoria?

Supplementary table 18. Design table 1 – manipulation checks.

Question	Hypothesis	Outcome measures	Sampling plan (e.g. power analysis)	Analysis Plan	Interpretation given to different outcomes	Outcome
**Zygomaticus Manipulation check 1.1:**<br>**Do participants without dysphoria smile when remembering positive memories?**	**Manipulation check 1.1 – Hypothesis 1:**<br>Individuals without dysphoria smile more when reliving positive memories than during baseline. $\gamma_2 > \gamma_1$	Zygomaticus major activity prior to baseline-correction.	The sampling plan is not determined by the manipulation check but by Test 1 and 2A.	We will employ Bayesian Informative Hypothesis Testing (BAIT)[63] which allows to test evidence for multiple hypotheses simultaneously. Only data for positive memories are included in this analysis. Analyses will be performed in three steps.<br>First, we will estimate a simple multilevel model that includes the dependent variable of interest, fixed effects for the predictors of interest, and a random intercept for participant to account for the within-participants design. The affective response of participant *i* to memory *j* is:	The manipulation with regard to positive autobiographical memory retrieval was successful. Individuals without dysphoria smile when they remember positive memories. It is possible to investigate the psychophysiological expression of positive affect during autobiographical memory retrieval.	Hypothesis 1 was confirmed.<br>The manipulation with regard to positive autobiographical memory retrieval was successful. Individuals without dysphoria smile when they remember positive memories. It is possible to investigate the psychophysiological expression of positive affect during autobiographical memory retrieval.
	**Manipulation check 1.1 – Hypothesis 2:**<br>Individuals without dysphoria smile less when reliving positive memories than during baseline. $\gamma_2 < \gamma_1$				The manipulation was not successful. Individuals without dysphoria smile less when remembering positive memories than during baseline. It is not possible to investigate the psychophysiological expression of positive affect during	

				$\text{affective\_response}_{ij} = \gamma_1(time)_{1i} + \gamma_2(time)_{2i} + \varepsilon_{ij} + U_i$<br><br>With:<br>$(time)_{1i} = 1$ if baseline<br>$(time)_{1i} = 0$ if recall<br>$(time)_{2i} = 0$ if baseline<br>$(time)_{2i} = 1$ if recall<br><br>*affective_response* represents zygomaticus activity, prior to transforming it into percentage change from baseline. The residual is indicated by ε_{ij}. $(time)$ represents the factor baseline versus recall. U_i represents a random intercept of a participant.<br><br>Second, we will extract the estimated effects of $(time)$ from the multilevel model as well as the variance-covariance matrix of these effects.<br><br>Third, the estimates and variance-	autobiographical memory retrieval.	
	**Manipulation check 1.1 – Null Hypothesis :**<br><br>Individuals without dysphoria smile equally when reliving positive memories and during baseline.<br>$\gamma_2 = \gamma_1$				The manipulation was not successful. Individuals without dysphoria smile equally when they remember positive memories compared to a baseline. It is not possible to investigate the psychophysiological expression of positive affect during autobiographical memory retrieval.	

				covariance matrices will be used to evaluate the evidence for each hypothesis with BAIT. For each analysis, we will calculate the effective sample size that accounts for multiple observations within participants. We will conduct all analyses with the default settings implemented in the bain package[63] with the exception that we will use a moderate fraction = 2 of the data to define the prior variance. We conduct sensitivity analyses with a more conservative fraction = 1 and a more liberal fraction = 3 to evaluate the influence of the prior variance on our results[63]. Evidence will be presented as Bayes Factors (BF) and Posterior Model Probabilities (PostP). The dependent variable is zygomaticus activity in positive memory trials, prior to baseline-correction.<br><br>γ_1 = individuals		

				without dysphoria: zygomaticus activity during baseline;<br>γ_2 = individuals without dysphoria: zygomaticus activity during memory retrieval<br><br>Since individuals with dysphoria are expected to experience affective memory distortions, the effectiveness of our manipulation will only be evaluated within the non-dysphoric group. However, we will conduct the same analyses within the dysphoric group to draw a comprehensive picture of how individuals with and without dysphoria re-experience autobiographical memories.		
**Zygomaticus Manipulation check 1.2:**<br><br>**Do participants without dysphoria smile more when remembering positive memories**	**Manipulation check 1.2 – Hypothesis 1:**<br><br>Individuals without dysphoria smile more when reliving positive memories than when remembering neutral memories.	Zygomaticus major activity after baseline correction.	The sampling plan is not determined by the manipulation check but by Test 1 and 2A.	We will employ Bayesian Informative Hypothesis Testing (BAIT)[63]. Only data of positive and neutral memories are included in this analysis. We will use a similar three step procedure as in	The manipulation was successful. Smiling during memory retrieval is specific for positive memories. It is possible to investigate the psychophysiological	Hypothesis 1 was confirmed.<br><br>The manipulation was successful. Smiling during memory retrieval is specific for positive memories. It is

**compared to neutral memories?**	$\gamma_2 > \gamma_1$			manipulation check 1, with the exception that $(time)$ is replaced by $(condition)$ with the levels positive and neutral. affective_response$_{\mathrm{ij}}$ is the percentage change of zygomaticus activity from baseline:<br>$\text{affective\_response}_{\mathrm{ij}} = \gamma_1(cond)_{1i} + \gamma_2(cond)_{2i} + \varepsilon_{\mathrm{ij}} + U_i$<br><br>With:<br>$(cond)_{1i} = 1$ if positive<br>$(cond)_{1i} = 0$ if neutral<br>$(cond)_{2i} = 0$ if positive<br>$(cond)_{2i} = 1$ if neutral<br><br>γ_1 = individuals without dysphoria: baseline-corrected zygomaticus activity during positive memory retrieval;<br>γ_2 = individuals without dysphoria: baseline-corrected zygomaticus activity	expression of positive affect during autobiographical memory retrieval.	possible to investigate the psychophysiological expression of positive affect during autobiographical memory retrieval.
	**Manipulation check 1.2 – Hypothesis 2:**<br><br>Individuals without dysphoria smile less when reliving positive memories than when remembering neutral memories.<br>$\gamma_2 < \gamma_1$				The manipulation was not successful. It is not possible to investigate the psychophysiological expression of positive affect during autobiographical memory retrieval.	
	**Manipulation check 1.2 – Null Hypothesis :**<br><br>Individuals without dysphoria smile equally when reliving positive memories and neutral memories.<br>$\gamma_2 = \gamma_1$				The manipulation was not successful. Smiling during memory retrieval is not specific for positive memories. It is not possible to investigate the psychophysiological expression of positive affect during autobiographical memory retrieval.	

				during neutral memory retrieval<br><br>Since individuals with dysphoria are expected to experience affective memory distortions, the effectiveness of our manipulation will only be evaluated within the non-dysphoric group. However, we will conduct the same analyses within the dysphoric group to draw a comprehensive picture of how individuals with and without dysphoria re-experience autobiographical memories.		
**Corrugator Manipulation check 2.1:**<br><br>**Do participants without dysphoria frown when remembering negative memories?**	**Manipulation check 2.1 – Hypothesis 1:**<br><br>Individuals without dysphoria frown more when reliving positive memories than during baseline. $\gamma_2 > \gamma_1$	Corrugator supercilii activity prior to baseline correction.	The sampling plan is not determined by the manipulation check but by Test 1 and 2A.	Similar analysis plan to Manipulation check 1.1, with the exception that *affective_response* represents corrugator activity instead of zygomaticus activity. Only data for negative memories are included in this analysis.<br><br>γ_1 = individuals without dysphoria: baseline-corrected	The manipulation with regard to negative autobiographical memory was successful. Individuals without dysphoria frown when they remember negative memories. It is possible to investigate the psychophysiological expression of negative affect during	Hypothesis 1 was confirmed.<br><br>The manipulation with regard to negative autobiographical memory was successful. Individuals without dysphoria frown when they remember negative memories. It is possible to investigate the

				corrugator activity during baseline; γ_2 = individuals without dysphoria: baseline-corrected corrugator activity during memory retrieval<br><br>Since individuals without dysphoria are expected to experience emotional memory distortions, the effectiveness of our manipulation will only be evaluated within the non-dysphoric group. However, we will conduct the same analyses within the dysphoric group to draw a comprehensive picture of how individuals with and without dysphoria re-experience autobiographical memories.	autobiographical memory retrieval.	psychophysiological expression of negative affect during autobiographical memory retrieval.
	**Manipulation check 2.1 – Hypothesis 2:**<br><br>Individuals without dysphoria frown less when reliving positive memories than during baseline. $\gamma_2 < \gamma_1$				The manipulation was not successful. Individuals without dysphoria frown less when remembering negative memories than during baseline. It is not possible to investigate the psychophysiological expression of negative affect during autobiographical memory retrieval.	
	**Manipulation check 2.1 – Null Hypothesis :**<br><br>Individuals without dysphoria frown equally when reliving positive memories and during baseline. $\gamma_2 = \gamma_1$				The manipulation was not successful. Individuals without dysphoria frown equally when they remember negative memories compared to a baseline. It is not possible to investigate the psychophysiological expression of negative affect during autobiographical memory retrieval.	

**Corrugator Manipulation check 2.2:**<br>**Do participants without dysphoria frown more when remembering negative memories compared to neutral memories?**	**Manipulation check 2.2 – Hypothesis 1:**<br>Individuals without dysphoria frown more when reliving negative memories than when remembering neutral memories.<br>$\gamma_2 > \gamma_1$	Corrugator supercilii activity after baseline correction.	The sampling plan is not determined by the manipulation check but by Test 1 and 2A.	Similar analysis plan to Manipulation check 1.2, with the exception that *affective_response* represents the percentage change from baseline of corrugator instead of zygomaticus activity. Only data for negative and neutral memories are included in this analysis.<br>γ_1 = individuals without dysphoria: baseline-corrected corrugator activity during negative memory retrieval; γ_2 = individuals without dysphoria: baseline-corrected corrugator activity during neutral memory retrieval<br>Since individuals with dysphoria are expected to experience emotional memory distortions, the effectiveness of our manipulation will only be evaluated within the non-dysphoric group. However, we will conduct the same analyses within the	The manipulation was successful. Frowning during memory retrieval is specific for negative memories. It is possible to investigate the psychophysiological expression of negative affect during autobiographical memory retrieval.	Hypothesis 1 was confirmed.<br>The manipulation was successful. Frowning during memory retrieval is specific for negative memories. It is possible to investigate the psychophysiological expression of negative affect during autobiographical memory retrieval.
	**Manipulation check 2.2 – Hypothesis 2:**<br>Individuals without dysphoria frown less when reliving negative memories than when remembering neutral memories.<br>$\gamma_2 < \gamma_1$				The manipulation was not successful. It is not possible to investigate the psychophysiological expression of negative affect during autobiographical memory retrieval.	
	**Manipulation check 2.2 – Null Hypothesis:**<br>Individuals without dysphoria frown equally when reliving negative memories and neutral memories.<br>$\gamma_2 = \gamma_1$				The manipulation was not successful. Frowning during memory retrieval is not specific for negative memories. It is not possible to investigate the psychophysiological expression of negative affect during	

				dysphoric group to draw a comprehensive picture of how individuals with and without dysphoria re-experience autobiographical memories.	autobiographical memory retrieval.	

Supplementary table 19. Design table 2 – Confirmatory analyses – Test 1 – Episodic detail.

Question	Hypothesis	Outcome measures	Sampling plan (e.g. power analysis)	Analysis Plan	Interpretation given to different outcomes	Outcome
**Test 1 - episodic detail:**<br>**Which theory best explains episodic memory distortions in dysphoria?**	**Hypothesis 1: Overgeneral memory bias**<br><br>Individuals with dysphoria retrieve fewer episodic detail when reliving positive memories and when reliving negative memories compared to individuals without dysphoria.<br>$\gamma_1 < \gamma_3$ & $\gamma_2 < \gamma_4$	Episodic memory detail.	We will collect data until there is strong evidence for one hypothesis ($PostP_i \geq .80$) or when we reach a maximum sample size of $N = 80$. We will commence by including a minimum of 20 participants per group (N=40) and computing Posterior Model Probabilities for Test 1 and Test 2A. If there is convincing evidence for a specific hypothesis within both tests ($PostP_i \geq .80$), we will stop data collection. Otherwise, we will increase the sample size in incremental steps of N=10 (5 per group) and repeat the BF_{ac} testing procedure each time.	We will employ Bayesian Informative Hypothesis Testing (BAIT)[63]. Analyses will be performed in three steps.<br><br>First, we will estimate a simple multilevel model that includes the dependent variable of interest, fixed effects for the predictors of interest, and a random intercept for participant to account for the within-participants design.<br><br>The episodic detail of participant i of memory j is:<br>$\text{episodic\_detail}_{ij} = \gamma_1(group\_cond)_{1i} + \gamma_2(group\_cond)_{2i} + \gamma_3(group\_cond)_{3i} + \gamma_4(group\_cond)_{4i} + \varepsilon_{ij} + U_i$<br><br>If dysphoric, positive memory:	An overgeneral memory bias best explains episodic memory distortions in individuals with dysphoria.	Hypothesis 2 was confirmed.<br>A negativity bias best explains episodic memory distortions in individuals with dysphoria.
	**Hypothesis 2: Negativity bias**<br><br>Individuals with dysphoria retrieve fewer episodic detail when reliving positive memories but more episodic detail when reliving negative memories compared to individuals without dysphoria.<br>$\gamma_1 < \gamma_3$ & $\gamma_2 > \gamma_4$				A negativity bias best explains episodic memory distortions in individuals with dysphoria.	

			At *N*=80, the results will be reported regardless of the strength of evidence for each hypothesis.	$(group\_cond)_{1i} = 1$<br>else<br>$(group\_cond)_{1i} = 0$<br><br>If dysphoric, negative memory:<br>$(group\_cond)_{2i} = 1$<br>else<br>$(group\_cond)_{2i} = 0$<br><br>If non-dysphoric, positive memory:<br>$(group\_cond)_{3i} = 1$<br>else<br>$(group\_cond)_{3i} = 0$<br><br>If non-dysphoric, negative memory:<br>$(group\_cond)_{4i} = 1$<br>else<br>$(group\_cond)_{4i} = 0$<br><br>The residual is indicated by ε_{ij}. U_i represents a random intercept of a participant.<br><br>Second, we will extract the estimated effects of interest from the multilevel model as well as the variance-covariance matrix of these effects.<br><br>Third, the estimates and variance-covariance matrices will be used to		
	**Hypothesis 0: Null hypothesis**<br><br>Individuals with dysphoria retrieve the same amount of episodic detail when reliving positive and negative memories compared to individuals without dysphoria.<br>$\gamma_1 = \gamma_3$ & $\gamma_2 = \gamma_4$				There are no differences in retrieved episodic detail during autobiographical memory recall between individuals with and without dysphoria.	
	**Complement hypothesis: Fail safe**<br><br>None of the other hypotheses explains the data well.				None of the above hypotheses explains the data well. In this case, exploratory analyses allow to generate hypotheses for future research.	

				evaluate the evidence for each hypothesis with BAIT. For each analysis, we will calculate the effective sample size that accounts for multiple observations within participants. We will conduct all analyses with the default settings implemented in the bain package[63] with the exception that we will use a moderate fraction = 2 of the data to define the prior variance. We conduct sensitivity analyses with a more conservative fraction = 1 and a more liberal fraction = 3 to evaluate the influence of the prior variance on our results[63]. Evidence will be presented as Bayes Factors (BF) and Posterior Model Probabilities (PostP). The dependent variable is the amount of episodic detail that is retrieved while reliving autobiographical memories.<br><br>γ_1 = individuals with dysphoria: positive memories; γ_2 = individuals with dysphoria: negative memories;		

				γ_3 = individuals without dysphoria: positive memories; γ_4 = individuals without dysphoria: negative memories		

Supplementary table 20. Design table 3 – Confirmatory analyses – Test 2A and 2B – Affective responses

**Question**	**Hypothesis**	**Outcome measure**	**Sampling plan (e.g. power analysis)**	**Analysis Plan**	**Interpretation given to different outcomes**	**Outcome**
**Test 2A – affective responses:**<br><br>**Which theory best explains affective memory distortions in dysphoria?**	**Hypothesis 1: Overgeneral memory bias**<br><br>Individuals with dysphoria experience diminished positive affect when remembering positive memories and diminished negative affect when remembering negative memorie*s,* compared to individuals without dysphoria.<br>$\gamma_1 < \gamma_3$ & $\gamma_2 < \gamma_4$	Baseline-corrected zygomaticus major activity for positive memories and baseline-corrected corrugator supercilii activity for negative memories	Similar sampling plan as for Test 1.	Similar analysis plan as for Test 1, but with the dependent variable *affective_response* instead of *episodic detail.*<br><br>*affective_response* is assessed with the primary outcome variables percentage change from baseline in zygomaticus and corrugator activity for positive and negative memories, respectively. Moreover, we will repeat this analysis with self-reported memory valence as complementary outcome variable.<br><br>γ_1 = individuals with dysphoria.: positive	An overgeneral memory bias best explains affective memory distortions in individuals with dysphoria.	Hypothesis 2 was confirmed.<br><br>A negativity bias best explains affective memory distortions in individuals with dysphoria.
	**Hypothesis 2: Negativity bias**<br><br>Individuals with dysphoria experience diminished positive affect and enhanced negative affect.<br>$\gamma_1 < \gamma_3$ & $\gamma_2 > \gamma_4$				A negativity bias best explains affective memory distortions in individuals with dysphoria.	

	**Hypothesis 0: Null hypothesis**<br><br>Individuals with dysphoria experience normal positive affect and normal negative affect.<br>$\gamma_1 = \gamma_3$ & $\gamma_2 = \gamma_4$<br><br>**Complement hypothesis: Fail safe**<br><br>None of the other hypotheses explains the data well.			memories; γ_2 = individuals with dysphoria.: negative memories;<br>γ_3 = individuals without dysphoria: positive memories; γ_4 = individuals without dysphoria: negative memories	There are no differences in affective responses to autobiographical memories between individuals with and without dysphoria.<br><br>None of the above hypotheses explains the data well. In this case, exploratory analyses allow us to generate hypotheses for future research.	
**Test 2B – domain-specificity of affective responses:**<br>**Are the distorted affective responses to autobiographical memories specific to memories or already present during the encoding of events?**	**In case of evidence for Test 2A – Hypothesis 1 (overgeneral memory bias):**<br><br>**Test 2B – Hypothesis 1:** Individuals with dysphoria experience diminished affective responses to positive movies and diminished affective responses to negative movies, compared to individuals without dysphoria.<br>$\gamma_1 < \gamma_3$ & $\gamma_2 < \gamma_4$	Zygomaticus major activity for positive movie clips and corrugator supercilii activity for negative memories.	The sampling plan is determined by Test 1 and Test 2A, not by Test 2B.	We will employ Bayesian Informative Hypothesis Testing (BAIT)[63]. Analyses will be conducted in three steps similar to Test 2A.<br><br>First, we will estimate a simple multilevel model that includes the dependent variable of interest, fixed effects for the predictors of interest, and a random intercept for participant to account for the within-participants design.	For all potential analysis within Test 2B, the conclusions are similar.<br>If there is evidence for Hypothesis 1, individuals with dysphoria show a similar pattern of distorted affective responses to novel experiences as to memories.<br>If there is evidence for the complement hypothesis, the pattern of affective responses in	Hypothesis 1 was confirmed.<br>Individuals with dysphoria show a similar pattern of distorted affective responses to novel experiences as to memories.

	**Test 2B – Complement hypothesis:**<br>Any other pattern of affective responses explains the data better.<br><br>**In case of evidence for Test 2A – Hypothesis 2 (negativity bias):**<br><br>**Test 2B – Hypothesis 1:**<br>Individuals with dysphoria experience diminished affective responses to positive movies and enhanced affective responses to negative movies.<br>$\gamma_1 < \gamma_3$ & $\gamma_2 > \gamma_4$<br><br>**Test 2B – Complement hypothesis:**<br>Any other pattern of affective responses explains the data better.			This model will be the same as for Test 2A, with the exception that the primary outcome variable *affective_response* refers to percentage change from baseline of zygomaticus and corrugator activity while watching positive and negative movie clips, respectively (instead of responses to memories). Moreover, we will repeat this analysis with self-reported memory valence as complementary outcome variable.<br><br>γ_1 = individuals with dysphoria: positive movies; γ_2 = individuals with dysphoria: negative movies;<br><br>γ_3 = individuals without dysphoria:	dysphoria is different than the pattern of affective responses to memories.	

	**In case of evidence for Test 2A – Null Hypothesis or Test 2A – Complement:**<br><br>We will not perform Test 2B as a confirmatory analysis.			positive movies; γ_4 = individuals without dysphoria: negative movies		

831

Supplementary table 21. Design table 4 – Confirmatory analyses – Test 3 – Relationship of episodic detail and affective responses

Question	Hypothesis	Outcome measure	Sampling plan (e.g. power analysis)	Analysis Plan	Interpretation given to different outcomes	Outcome
**Test 3.1A – The relationship between episodic detail and affective responses to positive memories:**<br><br>**Does the amount of retrieved episodic detail predict affective responses to a positive autobiographical memory among individuals with and without dysphoria?**	**Hypothesis 1: Positive relationship**<br><br>Within individuals, more episodic detail during memory retrieval predicts stronger affective responses to positive memories.<br><br>Dysphoric group:<br>$\gamma_1 > 0$<br>Non-dysphoric group:<br>$\gamma_2 > 0$	Baseline-corrected zygomaticus activity in response to memories.	The sampling plan is not determined by Test 3, but by Test 1 and 2A.	We will employ Bayesian Informative Hypothesis Testing (BAIT)[63].<br>First, we will estimate a multilevel model with the following equation (1), where the affective response to a memory of participant *i* in group *j* is:<br><br>$affective\_response_{ij} = \beta_{1i}(group)_{1i} + \beta_{2i}(group)_{2i} + \beta_{3i}\overline{(episodic\_detail)}_{ij}(group)_{1i} + \beta_{4i}\overline{(episodic\_detail)}_{ij}(group)_{2i} + \gamma_{1i}(episodic\_detail)^c_{ij}\,(group)_{1i} + \gamma_{2i}(episodic\_detail)^c_{ij}\,(group)_{2i} + \varepsilon_{ij} + U_i$<br><br>With:<br>$(group)_{1i} = 1$ if dysphoric<br>$(group)_{1i} = 0$ if non-dysphoric<br>$(group)_{2i} = 0$ if dysphoric<br>$(group)_{2i} = 1$ if non-dysphoric	If γ_1 (or γ_2) is larger than zero, positive memories that are retrieved with more episodic detail elicit stronger affective responses, within individuals with dysphoria (or without dysphoria).	The null hypothesis was confirmed.<br><br>There was no relationship between episodic detail and the affective response to a positive memory, within individuals with and without dysphoria.
	**Hypothesis 2: Negative relationship**				If γ_1 (or γ_2) is smaller than zero, positive memories that are retrieved with more episodic detail elicit	

	Within individuals, more episodic detail predicts weaker affective responses to positive memories.<br><br>Dysphoric group:<br>$\gamma_1 < 0$<br>Non-dysphoric group:<br>$\gamma_2 < 0$			*affective_response* is assessed with the primary outcome variable percentage change from baseline in zygomaticus activity (while remembering positive memories). Additionally, we will repeat this analysis with self-reported memory valence as complementary outcome measure. The factor dysphoric versus non-dysphoric is represented as $(group)$. β_{1i} and β_{2i} represent the estimated $affective\_response$ to a memory in the dysphoric group and non-dysphoric group, respectively, if $\overline{(episodic\_detail)}_{ij}$ and $(episodic\_detail)^c_{ij}$ are zero. β_{3i} and β_{4i} represent the linear effects of $\overline{(episodic\_detail)}_{ij}$ on *affective_response* in the dysphoric and non-dysphoric group, respectively. γ_{1i} and γ_{2i} represent the linear effects of $(episodic\_detail)^c_{ij}$ on *affective_response* in the dysphoric and non-dysphoric group, respectively. The superscript *c* indicates that the variable $(episodic\_detail)$ is participant mean-centred. $\overline{(episodic\_detail)}$ represents the grand-mean centred mean for each individual. The residual is indicated by ε_{ij}. U_i represents a random intercept of a participant.<br><br>The relationship between episodic detail and affective response is tested	weaker affective responses, within individuals with dysphoria (or without dysphoria).	
	**Null Hypothesis: No relationship**<br><br>Within individuals, episodic detail does not predict affective responses to positive memories.<br><br>Dysphoric group:<br>$\gamma_1 = 0$<br>Non-dysphoric group:<br>$\gamma_2 = 0$				If γ_1 (or γ_2) is zero, there is no relationship between episodic detail and the affective response to a positive memory, within individuals with dysphoria (or without dysphoria).	

				separately for individuals with and without dysphoria.<br>The hypotheses focus on the relationship of variations of episodic detail and affective responses within participants (γs), but we will also test the relationship of episodic detail and affective responses between participants (βs).<br>The dependent variable is baseline-corrected zygomaticus activity.		
**Test 3.1B – The relationship between episodic detail and affective responses to positive memories:**<br>**Does the within-participant relationship between episodic detail and affective responses to positive memories differ between individuals with and without dysphoria?**	**Hypothesis 1: Decoupling of episodic and emotional memory components**<br>The relationship between episodic detail and affective response is smaller in the dysphoric compared to the non-dysphoric group.<br>$\gamma_1 > \gamma_2$	Baseline-corrected zygomaticus activity in response to memories.	The sampling plan is not determined by Test 3, but by Test 1 and 2A.	We will employ Bayesian Informative Hypothesis Testing (BAIT)[63]. The analysis plan is exactly the same as for Test 3.1A with the exception that the γ coefficients are not compared to zero, but to each other.<br>The hypotheses focus on the relationship of variations of episodic detail and affective responses within participants (γs), but we will also test the relationship of episodic detail and affective responses between participants (βs).	The relationship between episodic detail and affective responses to positive memories is smaller among individuals with dysphoria compared to individuals without dysphoria. The key problem that drives distorted affective responses in dysphoria might not be a result of a failure to retrieve episodic detail, but a decoupling of episodic retrieval and affective responses.	The null hypothesis was confirmed.<br>There was no difference between individuals with and without dysphoria in the absent relationship of affective responses and episodic detail when remembering positive memories.
	**Hypothesis 2: Enhanced coupling of episodic and emotional**				The relationship between episodic detail and affective responses to positive memories is larger among	

	**memory components**<br><br>The relationship between episodic detail and affective response is larger in the dysphoric compared to the non-dysphoric group.<br>$\gamma_1 < \gamma_2$				individuals with dysphoria compared to individuals without dysphoria. Enhanced coupling of episodic and emotional memory systems might contribute to the maladaptive memory distortions in dysphoria.	
	**Null Hypothesis:**<br><br>The relationship between episodic detail and affective response is similar in the dysphoric and the non-dysphoric group.<br>$\gamma_1 = \gamma_2$				The relationship between episodic detail and affective responses to positive memories is similar in individuals with and without dysphoria. Distorted affective responses to autobiographical memories might be the result of a failure to retrieve memories with high episodic detail among individuals with dysphoria (as opposed to an altered coupling of episodic and emotional memory systems).	

**Test 3.2A – The relationship between episodic detail and affective responses to negative memories:**<br>**Does the amount of retrieved episodic detail predict affective responses to a negative autobiographical memory among individuals with and without dysphoria?**	**Hypothesis 1: Positive relationship**<br><br>Within individuals, more episodic detail during memory retrieval predicts stronger affective responses to negative memories.<br><br>Dysphoric group: $\gamma_1 > 0$<br>Non-dysphoric group: $\gamma_2 > 0$	Baseline-corrected corrugator supercilii activity in response to memories.	The sampling plan is not determined by Test 3, but by Test 1 and 2A.	The analysis plan for 3.2A and 3.2B is exactly the same as the analysis plan for 3.1A and 3.2B, with the exception that *affective_response* is assessed with the primary outcome variable percentage change from baseline in corrugator activity (while remembering negative memories). Additionally, we will repeat this analysis with self-reported memory valence as complementary outcome measure.<br><br>The hypotheses focus on the relationship of variations of episodic detail and affective responses within participants (γs), but we will also test the relationship of episodic detail and affective responses between participants (βs).	If γ_1(or γ_2) is larger than zero, negative memories that are retrieved with more episodic detail elicit stronger affective responses, within individuals with dysphoria (or without dysphoria).	The null hypothesis was confirmed.<br><br>There was no relationship between episodic detail and the affective response to a negative memory, within individuals with and without dysphoria.
	**Hypothesis 2: Negative relationship**<br><br>Within individuals, more episodic detail predicts weaker affective responses to negative memories.				If γ_1 (or γ_2) is smaller than zero, negative memories that are retrieved with more episodic detail elicit weaker affective responses, within individuals with dysphoria (or without dysphoria).	

	Dysphoric group:<br>$\gamma_1 < 0$<br>Non-dysphoric group:<br>$\gamma_2 < 0$					
	**Null Hypothesis: No relationship**<br><br>Within individuals, episodic detail does not predict affective responses to negative memories.<br><br>Dysphoric group:<br>$\gamma_1 = 0$<br>Non-dysphoric group:<br>$\gamma_2 = 0$				If γ_1 (or γ_2) is zero, there is no relationship between episodic detail and the affective response to a negative memory, within individuals with dysphoria (or without dysphoria).	
**Test 3.2B – The relationship between episodic detail and affective responses to negative memories:**	**Hypothesis 1: Decoupling of episodic and emotional memory components**<br><br>The relationship between	Baseline-corrected corrugator supercilii activity in response to memories.	The sampling plan is not determined by Test 3, but by Test 1 and 2A.	The analysis plan for 3.2A and 3.2B is exactly the same as the analysis plan for 3.1A and 3.2B, with the exception that *affective_response* is assessed with the primary outcome variable percentage change from baseline in corrugator activity (while remembering negative memories). Additionally, we will repeat this analysis with self-reported memory	The relationship between episodic detail and affective responses to negative memories is smaller among individuals with dysphoria compared to individuals without dysphoria. The key problem that drives	The null hypothesis was confirmed.<br><br>There was no difference between individuals with and without dysphoria in the absent relationship of affective responses and episodic detail when

**Does the within-participant relationship between episodic detail and affective responses to negative memories differ between individuals with and without dysphoria?**	episodic detail and affective response is smaller in the dysphoric compared to the non-dysphoric group.<br>$\gamma_1 > \gamma_2$			valence as complementary outcome measure.<br>The hypotheses focus on the relationship of variations of episodic detail and affective responses within participants (γs), but we will also test the relationship of episodic detail and affective responses between participants (βs).	distorted affective responses in dysphoria might not be a result of a failure to retrieve episodic detail, but a decoupling of episodic retrieval and affective responses.	remembering negative memories.
	**Hypothesis 2: Enhanced coupling of episodic and emotional memory components**<br><br>The relationship between episodic detail and affective response is larger in the dysphoric compared to the non-dysphoric group.<br>$\gamma_1 < \gamma_2$				The relationship between episodic detail and affective responses to negative memories is larger among individuals with dysphoria compared to individuals without dysphoria. Enhanced coupling of episodic and emotional memory systems might contribute to the maladaptive memory distortions in dysphoria.	
	**Null Hypothesis:**<br><br>The relationship between episodic detail and affective				The relationship between episodic detail and affective responses to negative memories is similar individuals with dysphoria compared to individuals without	

	response is similar in the dysphoric and the non-dysphoric group. $\gamma_1 = \gamma_2$				dysphoria.. Distorted affective responses to autobiographical memories might be the result of a failure to retrieve memories with high episodic detail individuals with dysphoria (as opposed to an altered coupling of episodic and emotional memory systems).	
