# Supplementary material for: Bayesian evaluation of diverging theories of episodic and affective memory distortions in dysphoria
Source: Nat Commun. 2024 Feb 13;15:1320. doi: 10.1038/s41467-024-45203-4 (PMC10864297; doi:10.1038/s41467-024-45203-4)
Supplement: Supplementary file 2 — Reporting Summary [file 41467_2024_45203_MOESM2_ESM.pdf]

nature portfolio

Corresponding author(s): Sascha Duken, MSc
Vanessa van Ast, PhD

Last updated by author(s): Aug 29, 2023

# Reporting Summary

Nature Portfolio wishes to improve the reproducibility of the work that we publish. This form provides structure for consistency and transparency in reporting. For further information on Nature Portfolio policies, see our Editorial Policies and the Editorial Policy Checklist.

## Statistics

For all statistical analyses, confirm that the following items are present in the figure legend, table legend, main text, or Methods section.

| n/a | Confirmed | |
|---|---|---|
| ☐ | ☒ | The exact sample size (*n*) for each experimental group/condition, given as a discrete number and unit of measurement |
| ☐ | ☒ | A statement on whether measurements were taken from distinct samples or whether the same sample was measured repeatedly |
| ☒ | ☐ | The statistical test(s) used AND whether they are one- or two-sided<br>*Only common tests should be described solely by name; describe more complex techniques in the Methods section.* |
| ☐ | ☒ | A description of all covariates tested |
| ☒ | ☐ | A description of any assumptions or corrections, such as tests of normality and adjustment for multiple comparisons |
| ☐ | ☒ | A full description of the statistical parameters including central tendency (e.g. means) or other basic estimates (e.g. regression coefficient) AND variation (e.g. standard deviation) or associated estimates of uncertainty (e.g. confidence intervals) |
| ☒ | ☐ | For null hypothesis testing, the test statistic (e.g. *F*, *t*, *r*) with confidence intervals, effect sizes, degrees of freedom and *P* value noted<br>*Give P values as exact values whenever suitable.* |
| ☐ | ☒ | For Bayesian analysis, information on the choice of priors and Markov chain Monte Carlo settings |
| ☐ | ☒ | For hierarchical and complex designs, identification of the appropriate level for tests and full reporting of outcomes |
| ☒ | ☐ | Estimates of effect sizes (e.g. Cohen's *d*, Pearson's *r*), indicating how they were calculated |

*Our web collection on statistics for biologists contains articles on many of the points above.*

## Software and code

Policy information about availability of computer code

| | |
|---|---|
| Data collection | Stimuli were presented with Neurobs Presentation software (https://www.neurobs.com, version 21). The task code and stimuli to which no legal restrictions apply are made publicly available on the Open Science Frameowork (https://doi.org/10.17605/OSF.IO/Y7346) upon acceptance of the stage 2 registered report manuscript. |
| Data analysis | Data analyses were conducted in R (version 4.3.0) as implemented in RStudio (version 2023.06.1), using the packages bain_0.2.8 and nlme_3.1-162. The analysis code are made publicly available upon acceptance of the stage 2 report on OSF (https://doi.org/10.17605/OSF.IO/Y7346). The conversion from raw data to processable data was conducted with the custom in-house program VSRRP developed by the Technical Support Group Psychology of the University. The manuscript includes a code availability statement:<br><br>All analysis code for R is publicly (https://doi.org/10.0.68.197/OSF.IO/Y7346). Only the conversion from raw data to processible data is conducted with the custom in-house program VSRRP (developed by the Technical Support Group Psychology at the University of Amsterdam). The (C/C++) source code of VSRRP that executes this conversion is available upon request from the Technical Support Group Psychology at the University of Amsterdam (https://lab-fmg.uva.nl/contact/contact.html) because it is not property of the authors of this manuscript. |

For manuscripts utilizing custom algorithms or software that are central to the research but not yet described in published literature, software must be made available to editors and reviewers. We strongly encourage code deposition in a community repository (e.g. GitHub). See the Nature Portfolio guidelines for submitting code & software for further information.

# Data

Policy information about availability of data

All manuscripts must include a data availability statement. This statement should provide the following information, where applicable:

- Accession codes, unique identifiers, or web links for publicly available datasets
- A description of any restrictions on data availability
- For clinical datasets or third party data, please ensure that the statement adheres to our policy

All raw and processed data as well as study materials are publicly available on the Open Science Framework (https://doi.org/10.0.68.197/OSF.IO/Y7346) as open data with some exceptions: We will not share highly sensitive data such as audio recordings and transcriptions of the personal autobiographical memories. Personal memories often cannot be completely anonymized and sharing them online without restrictions would violate the participants' privacy. These data are stored on secured servers of the University of Amsterdam. Should other researchers need access to these sensitive data, they must make a formal request to the ethics committee of the University of Amsterdam. However, for transparency and reproducibility, we offer exemplary memories of participants who explicitly consented that their memories may be shared. We also do not share materials that are subject to copyright by third parties such as movie clips. For materials that cannot be shared, we provided detailed descriptions that allow to reproduce them.

# Research involving human participants, their data, or biological material

Policy information about studies with human participants or human data. See also policy information about sex, gender (identity/presentation), and sexual orientation and race, ethnicity and racism.

| | |
|---|---|
| Reporting on sex and gender | We report self-reported sex (in Dutch 'Geslacht'). Our sample exists of almost exclusively self-reported women ('Vrouw'), with two exceptions that reported to be men ('Man'). We did not compare self-reported women and men because it was not part of our research question and because our sample doesn't include enough data for such a comparison. We address this in a paragraph on limitations of our study in the discussion. |
| Reporting on race, ethnicity, or other socially relevant groupings | According to European data protection and privacy guidelines, researchers are only permitted to collect data that are relevant for their research project. Therefore, we do not report race, ethnicity, or other social groupings, which we did not consider important prior to collecting the data. There may be a sampling bias in terms of social groupings that could be assessed in future studies, but for the present study we assumed to investigate relatively universal processes (within the context of Dutch society) that do not differ considerably between such social groups. |
| Population characteristics | See below (Behavioural & social sciences study design) |
| Recruitment | Participants were recruited via an online portal for volunteers of the University of Amsterdam, via advertisements on social media, as well as through posters, and flyers. There may be some selection biases. For example, some people may be more liekly to participate in a study that asks to share feelings in memories than other people. Unintentionally, our sample also includes more self-reported female participants, which indicates a selection bias (female individuals may have been more likely to participate than male or non-binary individuals). |
| Ethics oversight | Ethics committee of the University of Amsterdam (2019-CP-10552). All participants provided written informed consent. That includes participants over the age of 16 but under 18, who are allowed to participate in studies at the University of Amsterdam without parental consent. |

Note that full information on the approval of the study protocol must also be provided in the manuscript.

# Field-specific reporting

Please select the one below that is the best fit for your research. If you are not sure, read the appropriate sections before making your selection.

☐ Life sciences ☒ Behavioural & social sciences ☐ Ecological, evolutionary & environmental sciences

For a reference copy of the document with all sections, see nature.com/documents/nr-reporting-summary-flat.pdf

# Behavioural & social sciences study design

All studies must disclose on these points even when the disclosure is negative.

| | |
|---|---|
| Study description | Quantitative experimental study combining data from self-reports and psychophysiological measures (in particular facial electromyography of the corrugator supercilii and the zygomaticus major). |
| Research sample | We recruited a dysphoric group that experienced depressive symptoms (score of 16 or higher on Beck's Depression Inventory) and a non-dysphoric group that experienced no depressive symptoms (score of 3 or lower on Beck's Depression Inventory).<br><br>A detailed description of other inclusion and exclusion criteria can be found in the 'Participants' section of the manuscript: "we excluded participants who (1) self-reported a prior depressive episode, self-help for depression, or professional help for depression (e.g., counselling, antidepressant medication), (2) had a current or past diagnosis of a mental disorder, (3) had a current or past neurological illness or injury, (4) had a history of a traumatic experience that still affected them, (5) consumed more than 14 |

| | |
|---|---|
| | alcoholic drinks per week, (6) consumed recreative drugs more than once per week, (7) used psychoactive drugs (e.g., anxiolytics), (8) had significant visual or hearing impairments that couldn't be corrected, (9) were younger than 16 or older than 35, and (10) did not speak Dutch. By excluding participants who reported a depressive episode or professional help for depression in the past, we aimed to achieve a relatively homogenous sample of participants with dysphoria that experienced prototypic cognitive and affective distortions that were not yet affected by prior depressive episodes or professional interventions."<br><br>Even though we also aimed to recruit male participants, our sample included almost exclusively young self-reported female participants. However, mood disorders are particularly prevalent in women and often arise during adolescence or young adulthood. Therefore, our sample represents a population for which it is particularly important to understand affective and cognitive distortions that contribute to depressive symptoms. Nonetheless, the limitation regarding generalizability is mentioned in the discussion. |
| Sampling strategy | We employed a convenience sampling strategy. Participants were recruited via an online portal for volunteers of the University of Amsterdam, via advertisements on social media, as well as through posters, and flyers. Most participants were recruited through social media advertisements. We employed Bayesian Updating and planned to collect data until there was convincing evidence for one hypothesis relative to all other hypotheses under investigation (PostPi ≥ .80) for each of the primary research questions (Test 1 and Test 2A), or until we reached a maximum sample size of N = 80. A detailed description of the sampling strategy is presented in the 'Ethics Information' of the methods section and in the 'Participants' paragraph in the results section. |
| Data collection | We collected data in two experimental sessions in in the Behavioral Science Lab of the University of Amsterdam (https://lab-fmg.uva.nl/en). We combined a range of instruments and measures, including pen and paper questionnaires, behavioral data from computer tasks, and psychophysiological recordings (most importantly facial electromyography of the zygomaticus major and corrugator supercilii). The experimenter were blind to the participants' condition but not to the study hypotheses. There was no other person present in the lab except the experimenter and the participant.<br>A description of the data collection is presented in the Methods section. |
| Timing | Data of the first participant was collected on 15/06/2021. Data of the last participant was collected on 04/08/2022. |
| Data exclusions | One hundred thirty-seven participants attended the first session of the study after being included based on the online screening. Three participants did not return for the second session and we excluded 54 participants that did not meet all inclusion criteria after participation (e.g., because their BDI score changed between online screening and the second session; see Supplement 3, Supplementary Fig. 4, for an overview of the screening and inclusion procedure). The final sample consisted of n = 40 participants in the non-dysphoric group and n = 40 participants in the dysphoric group (N = 80).<br>Participants and trials were excluded following strict preregistered exclusion criteria. |
| Non-participation | Three participants did not return for the second session after having been tested for the first session. No other participant declined participation or dropped out. |
| Randomization | Potential participants completed an online screening questionnaire via the survey tool Qualtrics (Qualtrics, Provo, UT). The screening included the BDI-II to allocate participants to the dysphoric or non-dysphoric group as well as questions regarding other exclusion criteria such as excessive drug use (see Sampling Plan). Participants who reported a BDI-II below 4 (non-dysphoric group) or above 15 (dysphoric group) were invited to participate in the lab as soon as possible. Participants who met an exclusion criterion were not invited to the lab.<br>During session 1, participants completed a screening questionnaire that again assessed all exclusion criteria. If a participant meet any exclusion criterion, they were not tested further but compensated for the first session.<br>The non-dysphoric group was matched with the dysphoric group by age (± 2 years) and self-reported sex. We verified the allocation to the dysphoric and non-dysphoric with a pen and paper version of the BDI-II in the beginning of Session 2, because the BDI-II has been validated as a pen and paper questionnaire and because dysphoric symptoms might have changed in the time between the screening and Session 2. Participants whose BDI-II score was no longer 3 or lower or 16 or higher were excluded from the analyses and data of an additional participant was collected instead. |

# Reporting for specific materials, systems and methods

We require information from authors about some types of materials, experimental systems and methods used in many studies. Here, indicate whether each material, system or method listed is relevant to your study. If you are not sure if a list item applies to your research, read the appropriate section before selecting a response.

## Materials & experimental systems

| n/a | Involved in the study |
|---|---|
| ☒ | ☐ Antibodies |
| ☒ | ☐ Eukaryotic cell lines |
| ☒ | ☐ Palaeontology and archaeology |
| ☒ | ☐ Animals and other organisms |
| ☒ | ☐ Clinical data |
| ☒ | ☐ Dual use research of concern |
| ☒ | ☐ Plants |

## Methods

| n/a | Involved in the study |
|---|---|
| ☒ | ☐ ChIP-seq |
| ☒ | ☐ Flow cytometry |
| ☒ | ☐ MRI-based neuroimaging |
